# Multi-index spatio-temporal joint evaluation of traditional Chinese medicine service capacity: empirical analysis of china provincial panel data from 2012 to 2023

**DOI:** 10.3389/fpubh.2026.1884758

**Published:** 2026-07-28

**Authors:** Rui Quan, Shuang Ma, Yanhui Li, Liujie Fu, Jinyu Wu, Ruifeng Li

**Affiliations:** 1 Beijing University of Chinese Medicine, Beijing, China; 2 School of Management, Beijing University of Chinese Medicine, Beijing, China

**Keywords:** China, Dagum Gini coefficient, health equity, regional disparities, resource allocation, service capacity, Traditional Chinese Medicine

## Abstract

**Objective:**

To examine the spatiotemporal evolution and equity of traditional Chinese medicine (TCM) service capacity across China from 2012 to 2023 and to provide empirical evidence for improving regional resource allocation.

**Methods:**

Using data on TCM services from the China Health Statistical Yearbook (2013–2024 editions), we constructed a panel dataset for 31 provinces in China covering 2012–2023. Six core indicators were included: outpatient and emergency visits to TCM institutions, number of discharged patients, number of TCM medical and health institutions, number of beds, number of TCM practitioners, and number of staff in TCM hospitals. To improve interprovincial comparability, four contextual indicators were additionally incorporated: gross domestic product per capita, year-end resident population, administrative area, and population density. An entropy-weighting method was used to calculate the composite score of TCM service capacity for each province and year. Spatial dependence was assessed using global Moran's *I* based on an economic-geographic weight matrix and further examined through local hot spot analysis. Overall disparities, intra-regional disparities, inter-regional disparities, and the contribution of transvariation density were evaluated using the Dagum Gini coefficient and its decomposition.

**Results:**

From 2012 to 2023, TCM service capacity in China improved steadily. Outpatient and emergency visits, number of discharged patients, number of TCM medical and health institutions, number of beds, number of TCM practitioners, and number of staff in TCM hospitals increased by 105.5%, 99.0%, 135.0%, 145.4%, 114.0%, and 121.2%, respectively. Composite scores rose in all 31 provinces, with Beijing and Shanghai consistently ranking among the top-performing regions, while Sichuan, Chongqing, Guangdong, and Shandong showed particularly marked improvement. Spatial analysis indicated that global Moran's *I* was positive and statistically significant in every year, ranging from 0.140 to 0.224, suggesting a stable positive spatial autocorrelation in provincial TCM service capacity. Local spatial patterns were characterized by strengthening hot spots in northern China and relatively stable cold spots in the south. The Dagum Gini coefficient showed that overall national disparities declined from 0.2107 in 2012 to 0.1459 in 2023, with a mean value of 0.1767, indicating an overall convergence trend. Intra-regional disparities were relatively higher in the eastern and western regions and lower in the central and northeastern regions. Inter-regional disparities accounted for the largest share of overall inequality, with a mean contribution rate of 42.57%, exceeding that of intra-regional disparities (28.15%) and transvariation density (29.28%).

**Conclusion:**

From 2012 to 2023, TCM service capacity improved overall across provinces in China, although interprovincial disparities persisted. Overall inequality showed a converging trend and was accompanied by significant spatial clustering. In terms of the sources of disparity, inter-regional differences remained the primary driver of overall inequality, with particularly pronounced gradients between the eastern region and other parts of the country, whereas within-region disparities were relatively small in the central and northeastern regions. Future efforts should therefore focus not only on continued capacity expansion, but also on strengthening cross-regional coordination, optimizing the targeted allocation of resources, and promoting a more spatially balanced distribution of TCM services to improve equity in service capacity.

## 1 Introduction

Equity in the allocation of health resources is a core concern in public health because it shapes the accessibility, efficiency, and quality of health services (1–4). In a country as geographically extensive and socioeconomically diverse as China, regional disparities in health resource distribution remain substantial, reflecting differences in economic development, population structure, transportation conditions, and health system capacity (5–7). The National Health Plan for the 14th Five-Year Plan explicitly emphasizes strengthening the public health protection network and improving the healthcare service system, underscoring a policy commitment to narrowing regional gaps and promoting a more equitable distribution of medical resources. Within this policy context, examining how healthcare resources are distributed across space and how these patterns evolve over time is important for both evidence-based decision-making and policy evaluation (3, 8).

As a distinctive and longstanding component of China's healthcare system, traditional Chinese medicine (TCM) plays an important role in disease prevention, treatment, rehabilitation, and health maintenance (9, 10). With the implementation of the Healthy China strategy and the growing emphasis on integrated care, TCM has assumed an increasingly visible role in expanding service provision and supporting population health (10, 11). In some areas, especially those with limited access to high-level biomedical services, TCM institutions function as an important complement to general healthcare. Optimizing the allocation of TCM resources is therefore relevant not only to the development of the TCM sector itself, but also to the broader goals of strengthening health system resilience and improving service inclusiveness (9).

Previous studies have examined regional differences in TCM development from multiple perspectives, including the distribution of institutions, workforce availability, service utilization, and institutional capacity (12–14). Existing evidence suggests that sizable provincial disparities persist in TCM personnel and service capacity and that these disparities are associated with broader socioeconomic and geographic conditions (12–14). Related research on health resource allocation in China has further shown that regional inequality is shaped by economic development, demographic characteristics, and policy support (5–8, 15). In addition, an increasing number of studies have introduced spatial approaches into the evaluation of health inequities, highlighting the importance of spatial dependence and geographic heterogeneity in understanding resource allocation patterns (16–18).

Despite these contributions, several limitations remain. First, much of the existing literature relies on cross-sectional comparisons and is therefore less able to capture long-term changes in TCM resource allocation (12, 13). Second, although inequity in healthcare resources is inherently spatial, the spatiotemporal distribution and evolution of TCM service capacity have not been sufficiently explored. Third, previous studies have largely evaluated traditional Chinese medicine (TCM) service capacity using single resource-based indicators, with limited attention to a more comprehensive perspective that simultaneously captures resource supply, service utilization, and regional contextual conditions (12). Fourth, although existing studies have increasingly incorporated spatial perspectives, limited attention has been paid to the spatial autocorrelation of TCM service capacity, the evolution of local hot and cold spots, and their relationship with the decomposition of regional disparities. Previous research has examined regional differences, dynamic evolution, and spatial spillover effects in the allocation efficiency of medical resources in TCM hospitals (19). However, studies that approach this issue from a comprehensive TCM service capacity perspective and integrate spatial autocorrelation analysis with Dagum Gini coefficient decomposition remain insufficient. Therefore, it is necessary to establish a comprehensive analytical framework that integrates spatial correlation analysis with Dagum Gini coefficient decomposition to systematically investigate the spatiotemporal evolution of TCM service capacity and the sources of its disparities.

To address these gaps, this study included 31 provincial-level administrative regions in China from 2012 to 2023, excluding Hong Kong, Macao, and Taiwan. Despite the continued expansion of traditional Chinese medicine (TCM) services in China, the temporal evolution, spatial distribution, and sources of regional disparities in service capacity remain insufficiently understood. To address this gap, provincial TCM service capacity was assessed using a composite index based on six core indicators—outpatient and emergency visits, number of discharged patients, number of institutions, number of beds, number of TCM practitioners, and number of staff in TCM hospitals—while accounting for regional contextual factors, including gross domestic product per capita, resident population, administrative area, and population density. The entropy-weighting method was used to generate province-level composite scores, and spatial autocorrelation analyses based on an economic-geographic weight matrix were applied to identify global and local clustering of high- and low-value areas. In addition, the Dagum Gini coefficient was used to decompose overall inequality into intra-regional differences, inter-regional differences, and transvariation density, thereby clarifying the spatiotemporal evolution and underlying sources of disparities in TCM service capacity across China (20). By combining multidimensional evaluation with inequality decomposition, this study aims to characterize the spatiotemporal evolution of TCM service capacity in China and to provide empirical evidence for improving the equity of regional health resource allocation.

## 2 Materials and methods

### 2.1 Data sources

Data on TCM service capacity were obtained from the TCM service section of the China Health Statistics Yearbook series for 2013–2024, including its earlier editions, the China Health Statistical Yearbook and the China Health and Family Planning Statistics Yearbook. A total of 31 provincial-level administrative regions in China were included for the period 2012–2023. Traditional Chinese medicine (TCM) service capacity was evaluated using six core indicators: outpatient and emergency visits, number of discharged patients, total number of TCM medical and health institutions, number of beds in TCM medical institutions, number of TCM practitioners, and number of staff in TCM hospitals. To enhance cross-provincial comparability, four regional covariates were also incorporated into the composite evaluation, including gross domestic product per capita, resident population at year-end, administrative area, and population density. A small proportion of missing data was imputed using mean substitution.

### 2.2 Composite evaluation of TCM service capacity

The 10 indicators were standardized and objectively weighted using the entropy weight method. Existing studies have assessed China's TCM medical service system from a comprehensive evaluation perspective, but most have relied on methods such as factor analysis and TOPSIS (14). Of these, six indicators captured traditional Chinese medicine (TCM) service capacity in terms of resource supply and service utilization, while four regional characteristic variables were incorporated to improve cross-provincial comparability. Following dimensionless transformation, weights were derived from the entropy values and combined to calculate annual composite scores of TCM service capacity for each province (21).

### 2.3 Spatial correlation analysis

Provincial composite scores of traditional Chinese medicine (TCM) service capacity were used to evaluate spatial dependence. Global Moran's *I* was estimated for the period 2012–2023 using an economic-geographic weight matrix that incorporated both geographic proximity and economic distance to test for spatial autocorrelation among provinces. The Getis–Ord Gi^*^ statistic was further applied to detect local spatial clustering and to characterize the spatial distribution and temporal evolution of hot spots (high-value clusters) and cold spots (low-value clusters). Moran's *I* values >0 indicate positive spatial autocorrelation, whereas values < 0 indicate negative spatial autocorrelation; statistical significance was assessed using *z*-scores and *p*-values.

### 2.4 Measurement of inequality

The Dagum Gini coefficient was used to assess the distributional equity of TCM service capacity across provinces (20). The Dagum Gini coefficient was used to assess interprovincial disparities in the composite scores of traditional Chinese medicine (TCM) service capacity across 31 provinces. Overall inequality was further decomposed into intraregional differences, interregional differences, and transvariation intensity. For regional comparison and decomposition, the provinces were classified into four regions according to China's official statistical grouping: eastern, central, western, and northeastern China.

### 2.5 Statistical analysis

A panel dataset for 2012–2023 was constructed in Excel 2013. Data cleaning, entropy weight estimation, and Dagum Gini coefficient decomposition were performed using Stata and SPSSAU. Global Moran's *I*, Moran scatter plots, and hot spot/cold spot spatial visualization were generated using ArcGIS and related software. The results are presented by integrating descriptive statistics, spatial analysis, and disparity decomposition (16–18).

## 3 Results

### 3.1 Growth in TCM service capacity

TCM service capacity in China expanded markedly between 2012 and 2023. Table 1 presents the overall status of TCM service capacity in China in 2012 and 2023. Outpatient and emergency visits to TCM medical institutions increased from 746.952 million to 1.535008 billion, representing a rise of 105.5%. Discharges from TCM medical institutions increased from 20,222,218 to 40,230,732, a rise of 99.0%. The number of TCM medical and health institutions increased from 39,382 to 92,531, corresponding to growth of 135.0%. The number of beds in TCM medical institutions grew from 705,795 to 1,731,946, representing the largest increase among the six indicators at 145.4%. Over the same period, the number of TCM practitioners increased from 488,367 to 1,045,000, and the number of staff in TCM hospitals increased from 818,775 to 1,810,814, with growth rates of 114.0% and 121.2%, respectively. We further calculated the per-1,000-population values for all six indicators. Although population growth over the 12-year period resulted in slightly lower growth rates for per-capita TCM service indicators than for the corresponding absolute counts, all six indicators continued to show an upward trend.

**Table 1:** Status of Traditional Chinese Medicine (TCM) service capacity in China in 2012 and 2023.

Class 1						
Indicator	Total			Per 1,000 population		
	2012	2023	Growth ratio	2012	2023	Growth ratio
Number of visits to traditional Chinese medicine (TCM) medical institutions (in ten thousand)	74,695.2	153,500.8	105.5%	0.06	0.11	83.3%
Number of patients discharged from medical institutions specializing in traditional Chinese medicine	202,22218	40,230,732	99.0%	14.90	28.58	91.8%
Number of medical and health institutions specializing in traditional Chinese medicine	39,382	92,531	135.0%	0.03	0.07	133.3%
Number of beds in medical institutions specializing in traditional Chinese medicine	705,795	1,731,946	145.4%	0.52	1.23	136.5%
Number of traditional Chinese medicine practitioners	488,367	1,045,000	114.0%	0.36	0.74	105.6%
Number of staff in a Traditional Chinese Medicine hospital	818,775	1,810,814	121.2%	0.60	1.29	115%

Taken together, these changes point to substantial expansion in both utilization and institutional capacity. The entropy weights of the six indicators were relatively balanced, indicating that each dimension contributed meaningfully to the composite evaluation.

### 3.2 Provincial variation in composite scores

**Figure 1:**
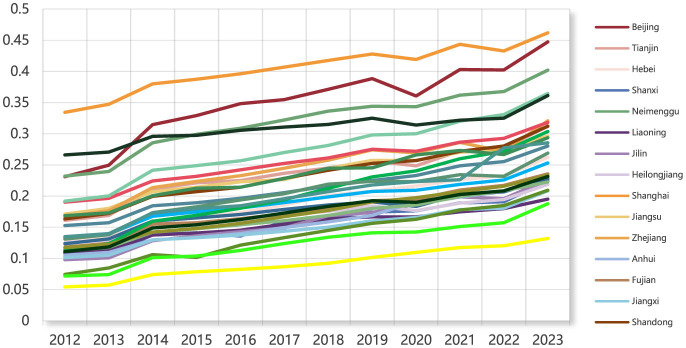
Status of TCM service capabilities among Chinese provinces from 2012 to 2023.

To improve comparability across provinces given differences in per capita resources, geographic size, and population, four province-level indicators—annual GDP per capita, year-end resident population, administrative area, and population density—were included in the entropy weight analysis alongside six per-1,000-population indicators of TCM service capacity. Table 2 reports the composite TCM service capacity scores for 31 provinces in China over 2012–2023. The current status of TCM service capacity across provinces in China from 2012 to 2023 is presented in Figure 1. Overall, provincial scores generally increased steadily over the study period, suggesting continued advancement of the TCM service system in China and a substantial overall improvement in provincial TCM service capacity.

**Table 2:** Entropy weight method scores for 31 provincial-level regions in China, 2012–2023.

Id	Area	2012	2013	2014	2015	2016	2017	2018	2019	2020	2021	2022	2023
1	Beijing	0.2310	0.2495	0.3145	0.3290	0.3483	0.3548	0.3713	0.3883	0.3607	0.4029	0.4024	0.4473
2	Tianjin	0.1610	0.1690	0.2113	0.2214	0.2250	0.2365	0.2450	0.2563	0.2485	0.2726	0.2763	0.3122
3	Hebei	0.1304	0.1337	0.1595	0.1656	0.1736	0.1859	0.2011	0.2078	0.2163	0.2264	0.2328	0.2623
4	Shanxi	0.1020	0.1061	0.1378	0.1411	0.1359	0.1593	0.1667	0.1743	0.1763	0.1890	0.1908	0.2245
5	Neimenggu	0.2321	0.2395	0.2856	0.2992	0.3089	0.3220	0.3362	0.3441	0.3435	0.3617	0.3677	0.4019
6	Liaoning	0.1086	0.1130	0.1374	0.1407	0.1453	0.1549	0.1633	0.1668	0.1666	0.1749	0.1798	0.1953
7	Jilin	0.0981	0.1010	0.1284	0.1362	0.1430	0.1487	0.1685	0.1739	0.1868	0.1986	0.1958	0.2267
8	Heilongjiang	0.1249	0.1297	0.1532	0.1562	0.1615	0.1705	0.1755	0.1841	0.1767	0.1886	0.1944	0.2189
9	Shanghai	0.3342	0.3472	0.3801	0.3875	0.3963	0.4069	0.4176	0.4280	0.4191	0.4435	0.4329	0.4619
10	Jiangsu	0.1711	0.1803	0.2070	0.2142	0.2230	0.2264	0.2442	0.2574	0.2572	0.2702	0.2747	0.2963
11	Zhejiang	0.1666	0.1767	0.2135	0.2235	0.2327	0.2447	0.2576	0.2740	0.2694	0.2861	0.2711	0.3207
12	Anhui	0.1050	0.1098	0.1299	0.1337	0.1399	0.1495	0.1586	0.1689	0.1912	0.1984	0.2075	0.2286
13	Fujian	0.1181	0.1241	0.1549	0.1573	0.1637	0.1715	0.1823	0.1924	0.1948	0.2097	0.2170	0.2355
14	Jiangxi	0.1011	0.1053	0.1296	0.1333	0.1376	0.1436	0.1503	0.1601	0.1658	0.1784	0.1805	0.2084
15	Shandong	0.1635	0.1722	0.1997	0.2070	0.2143	0.2286	0.2414	0.2524	0.2571	0.2718	0.2804	0.3121
16	Henan	0.1527	0.1574	0.1843	0.1893	0.1963	0.2056	0.2163	0.2249	0.2331	0.2485	0.2553	0.2804
17	Hubei	0.1237	0.1313	0.1598	0.1649	0.1706	0.1786	0.1859	0.1906	0.1835	0.2024	0.2069	0.2279
18	Hunan	0.1333	0.1392	0.1676	0.1746	0.1808	0.1895	0.1986	0.2075	0.2094	0.2188	0.2258	0.2532
19	Guangdong	0.1904	0.1965	0.2244	0.2316	0.2421	0.2525	0.2612	0.2752	0.2717	0.2863	0.2927	0.3182
20	Guangxi	0.1117	0.1179	0.1420	0.1477	0.1539	0.1606	0.1688	0.1801	0.1858	0.1994	0.2083	0.2228
21	Hainan	0.0543	0.0571	0.0740	0.0785	0.0824	0.0866	0.0923	0.1017	0.1097	0.1175	0.1204	0.1321
22	Chongqing	0.1140	0.1235	0.1596	0.1691	0.1807	0.1941	0.2124	0.2308	0.2406	0.2598	0.2718	0.3035
23	Sichuan	0.1919	0.2003	0.2415	0.2490	0.2568	0.2699	0.2813	0.2982	0.3001	0.3202	0.3307	0.3646
24	Guizhou	0.0743	0.0848	0.1061	0.1017	0.1212	0.1332	0.1451	0.1564	0.1628	0.1778	0.1849	0.2095
25	Yunnan	0.1173	0.1226	0.1425	0.1481	0.1562	0.1670	0.1770	0.1907	0.1974	0.2070	0.2158	0.2336
26	Xizang	0.1686	0.1730	0.2007	0.2127	0.2144	0.2282	0.2447	0.2454	0.2661	0.2730	0.2666	0.2935
27	Shaanxi	0.1109	0.1185	0.1489	0.1540	0.1624	0.1729	0.1835	0.1924	0.1900	0.2027	0.2084	0.2313
28	Gansu	0.1309	0.1383	0.1734	0.1827	0.1939	0.2040	0.2190	0.2237	0.2234	0.2343	0.2318	0.2688
29	Qinghai	0.1348	0.1399	0.1728	0.1799	0.1845	0.1959	0.2072	0.2179	0.2232	0.2261	0.2773	0.2854
30	Ningxia	0.0718	0.0742	0.1013	0.1040	0.1130	0.1238	0.1339	0.1410	0.1423	0.1511	0.1574	0.1878
31	Xinjiang	0.2661	0.2707	0.2958	0.2973	0.3054	0.3106	0.3149	0.3251	0.3141	0.3218	0.3251	0.3611

In terms of regional distribution, the eastern region generally remained at a relatively high level, reflecting stronger TCM service provision capacity and greater resource concentration. Shanghai and Beijing consistently ranked among the top provinces across all study years, with scores of 0.4619 and 0.4473, respectively, in 2023, indicating a marked overall advantage in TCM service capacity in first-tier cities. Guangdong, Zhejiang, Jiangsu, and Shandong also reported relatively high scores, further suggesting that TCM service capacity in the eastern region was generally strong and stable.

From a regional distribution perspective, the eastern region remained at a relatively high level overall, indicating stronger TCM service provision capacity and a greater concentration of resources. Shanghai and Beijing consistently ranked among the top-performing provinces throughout the study period, with scores of 0.4619 and 0.4473, respectively, in 2023. Guangdong, Zhejiang, Jiangsu, and Shandong also reported relatively high scores, further suggesting that TCM service capacity in the eastern region was generally strong and exhibited stable development.

In the central and western regions, some provinces exhibited relatively rapid improvement during the study period. For instance, the composite scores of Sichuan, Inner Mongolia, Xinjiang, and Tibet increased continuously, with Sichuan rising from 0.1919 in 2012 to 0.3646 in 2023, indicating a particularly notable increase. Guizhou, Ningxia, and Hainan, although starting from relatively low baseline levels, also showed sustained overall growth. These findings suggest that TCM service capacity in historically weaker regions has gradually improved, although substantial interprovincial disparities in development levels persist (6–8).

Notably, slight fluctuations were observed in a few provinces around 2020. Scores in Beijing, Shanghai, Zhejiang, and Xinjiang declined temporarily before returning to an upward trend. Overall, these short-term fluctuations did not alter the broader pattern of improvement in provincial TCM service capacity.

To test the robustness of the findings to the specification of regional characteristic variables, gross domestic product per capita, administrative area, population density, and year-end resident population were excluded as size-related control variables, and the allocation level of TCM medical and health resources was re-estimated for 31 provincial-level administrative regions in China over 2012–2023. The rankings obtained under the two specifications were compared for each year, and Spearman's rank correlation coefficient was employed to evaluate the consistency of the results.

As shown in Table 3, the Spearman rank correlation coefficients between provincial rankings generated by the two entropy weight method specifications were positive in all years during 2012–2023, ranging from 0.400 to 0.581, with an average of 0.500, indicating a moderate positive correlation overall. This suggests that, although the exclusion of selected indicators led to some fluctuations in provincial composite scores and specific rankings, the two evaluation schemes were generally consistent in identifying the relative ordering across provinces. Accordingly, the basic gradient pattern of TCM medical and health resource allocation in China did not change substantially, indicating that the main findings are robust.

**Table 3:** Spearman rank correlation coefficients of provincial rankings under two entropy weighting methods.

Year	Spearman rank correlation coefficient (ρ)	Degree of correlation
2012	0.410	Moderate positive correlation
2013	0.438	Moderate positive correlation
2014	0.529	Moderate positive correlation
2015	0.563	Moderately strong positive correlation
2016	0.574	Moderately strong positive correlation
2017	0.538	Moderate positive correlation
2018	0.581	Moderately strong positive correlation
2019	0.572	Moderately strong positive correlation
2020	0.418	Moderate positive correlation
2021	0.400	Moderate positive correlation
2022	0.459	Moderate positive correlation
2023	0.517	Moderate positive correlation
Average	0.500	Moderate positive correlation

Across years, the rank correlation coefficients were relatively high in 2015–2019, with all values above 0.538, suggesting a high degree of consistency between the rankings produced by the two entropy weight method specifications during this period. However, the coefficients decreased in 2020–2022, with the lowest value observed in 2021 (0.400). This indicates that, after removing regional characteristic variables such as economic development, spatial scale, and population density, the rankings of some provinces became more sensitive to variation in the core indicators, leading to greater disruption in local ordering under the alternative specification. Importantly, the coefficients remained positive throughout and stayed overall at a moderate level, suggesting that the observed differences mainly reflected local shifts in ranking rather than a fundamental reversal of the overall pattern.

Taken together, these findings suggest that excluding selected indicators reduced the contribution of regional characteristic variables and placed greater weight on the dispersion of the core resource indicators, thereby amplifying ranking fluctuations for some provinces in specific years. In contrast, the original entropy weight method preserved information on core resource allocation indicators while also incorporating regional differences in economic development, population, and spatial conditions, which likely helped smooth short-term anomalies and yielded more stable evaluation results. Overall, the Spearman rank correlation analysis supports the robustness and credibility of the main findings derived from the original entropy weight method. This finding is consistent with previous studies showing that the efficiency of medical resource allocation in TCM hospitals is characterized by spatial spillover effects (19).

### 3.3 Spatial correlation analysis

**Figure 2:**
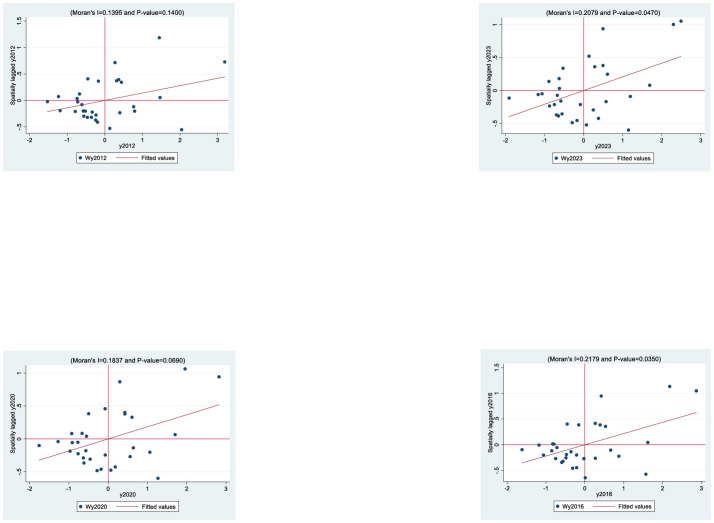
Moran Scatter Plots of Chinese Provinces in 2012, 2016, 2020, and 2023.

Based on the economic-geographic matrix, Table 4 reports the global Moran's I values for TCM service capacity across 31 provinces in China during 2012–2023. Figure 2 further provides a visual presentation of the spatial association characteristics in each year through Moran scatter plots.

**Table 4:** Moran's *I* under the economic-geographic weight matrix.

Variables	*I*	*E*(*I*)	sd(*I*)	*z*	*p*-value^*^
y2012	0.14	−0.033	0.094	1.832	0.033
y2013	0.16	−0.033	0.094	2.053	0.02
y2014	0.205	−0.033	0.096	2.494	0.006
y2015	0.213	−0.033	0.096	2.563	0.005
y2016	0.218	−0.033	0.096	2.618	0.004
y2017	0.214	−0.033	0.096	2.579	0.005
y2018	0.218	−0.033	0.096	2.612	0.004
y2019	0.224	−0.033	0.096	2.668	0.004
y2020	0.184	−0.033	0.097	2.247	0.012
y2021	0.214	−0.033	0.096	2.566	0.005
y2022	0.209	−0.033	0.097	2.503	0.006
y2023	0.208	−0.033	0.097	2.482	0.007

Overall, the global Moran's I remained positive throughout the study period, ranging from 0.140 to 0.224 and consistently exceeding the expected value of *E*(*I*) = −0.033. These findings indicate that provincial TCM service capacity in China was not randomly distributed, but exhibited a stable positive spatial correlation, with high-value provinces tending to cluster with other high-value provinces and low-value provinces tending to cluster with low-value provinces. Moran's *I* increased from 0.140 in 2012 to 0.224 in 2019, indicating a gradual strengthening of spatial dependence and increasingly pronounced interprovincial agglomeration effects. Although the index fell to 0.184 in 2020, it remained around 0.208 during 2021–2023, suggesting that despite short-term fluctuations associated with external shocks, the overall spatial association remained strongly positive.

The significance tests further showed that the z-statistics were positive for all years and that all *p*-values were < 0.05, indicating that the global Moran's *I* was statistically significant throughout the study period. The Moran scatter plots were consistent with these results, as the fitted line showed a generally positive slope and most observations were located in the first and third quadrants. These findings suggest that TCM service capacity in China demonstrates clear spatial spillover effects and strong regional linkage.

Building on the global Moran's *I* results, ArcGIS was used to visualize local spatial clustering patterns in 2012, 2016, 2020, and 2023, and hotspot-coldspot maps were generated. The labels in the figure correspond to these four years and are aligned with the Moran scatter plots presented earlier. Overall, the hotspot-coldspot distributions in all four years revealed clear local spatial clustering, suggesting that provincial TCM service capacity was not randomly distributed, but characterized by significant spatial heterogeneity and local spatial correlation. These findings are consistent with the positive spatial autocorrelation indicated by the global Moran's *I* analysis.

The evolution of hotspot areas showed that high-value clusters gradually shifted from a scattered to a more concentrated pattern and from weaker to stronger statistical significance over the study period. In 2012, Xinjiang was the only hotspot at the 90% confidence level, suggesting limited spatial linkage among high-value provinces. From 2016 onward, hotspot areas became increasingly concentrated in northern China and surrounding areas, with Beijing, Hebei, Shanxi, and Shandong all identified as hotspots at the 90% confidence level, indicating the emergence of local clustering among high-value provinces. In 2020, Beijing was identified as a hotspot at the 95% confidence level, while Hebei, Shanxi, Shandong, and Henan were identified as hotspots at the 90% confidence level, reflecting an increase in both clustering intensity and spatial coverage. By 2023, Beijing was identified as a hotspot at the 99% confidence level; Hebei, Shanxi, Shandong, and Henan at the 95% confidence level; and Ningxia at the 90% confidence level, indicating a further strengthening and spatial expansion of the high-value clustering pattern.

The distribution of coldspot areas showed that low-value clusters were mainly concentrated in Southwest and South China and remained relatively stable over the study period. In 2012, Chongqing, Guizhou, and Guangxi were identified as coldspots at the 90% confidence level, whereas Guangdong and Hainan were identified as coldspots at the 99% confidence level. This pattern largely persisted in 2016, indicating that a relatively stable local spatial association had formed among low-value provinces. In 2020, the coldspot area contracted slightly: Chongqing was no longer significant, Guizhou remained a coldspot at the 90% confidence level, and Guangxi, Guangdong, and Hainan continued to be identified as coldspots at the 99% confidence level. By 2023, Guizhou remained a coldspot at the 90% confidence level, while Guangxi, Guangdong, and Hainan continued to be identified as coldspots at the 99% confidence level. Overall, these results suggest that several provinces in Southwest and South China remained in a persistent low-value clustering state, showing strong spatial path dependence and lock-in effects.

**Figure 3:**
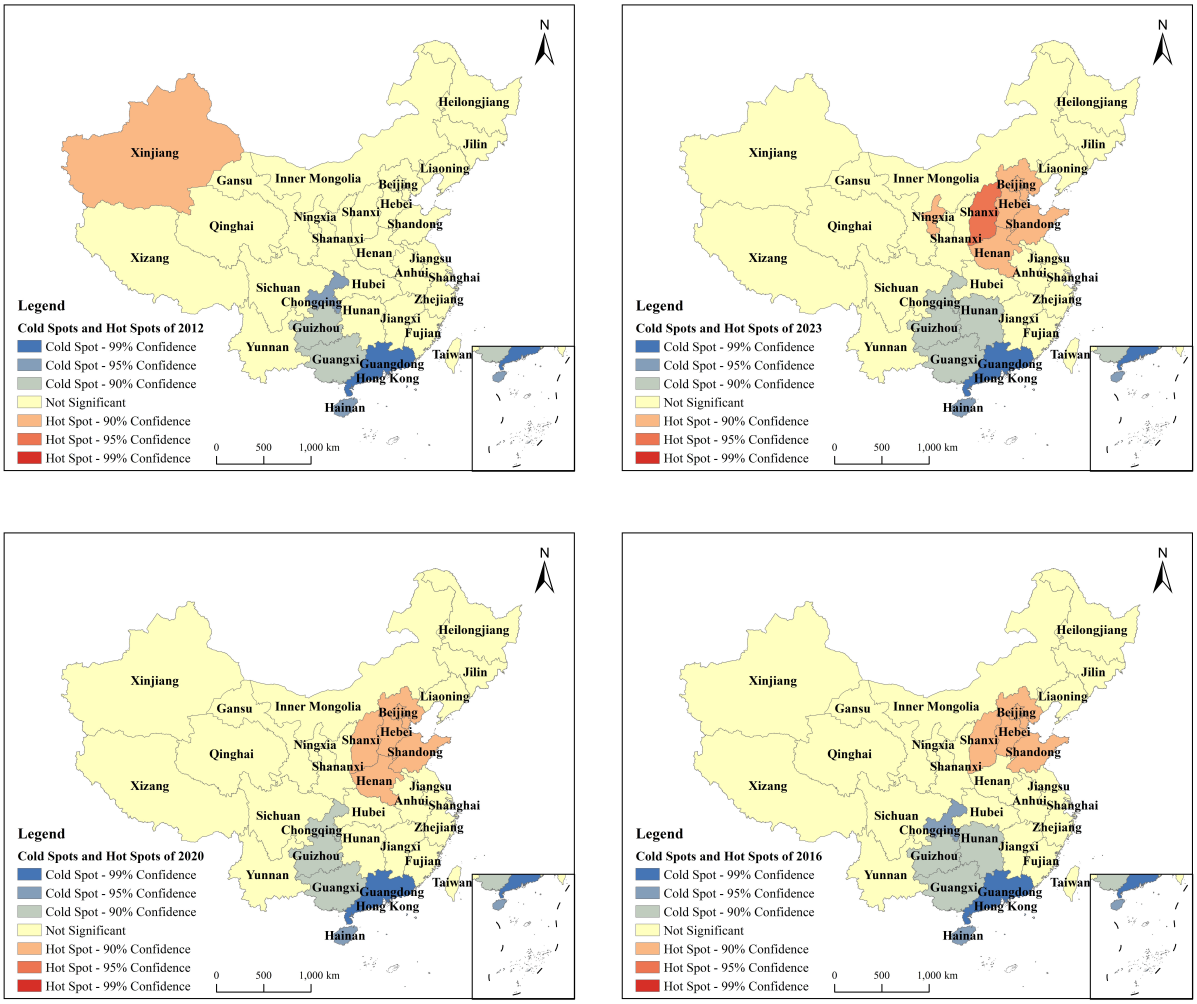
Spatial Distribution of Cold and Hot Spots Across Chinese Provinces in 2012, 2016, 2020, and 2023.

Overall, the local spatial pattern of the study object over the study period was characterized by strengthened hotspot clustering in the north and stable coldspot clustering in the south. High-value clusters gradually became concentrated in North China and surrounding areas, particularly in Beijing, Hebei, Shanxi, Shandong, and Henan, with increasing statistical significance over time. In contrast, low-value clusters remained relatively stable in Southwest and South China, especially in Guangxi, Guangdong, Hainan, and Guizhou. These findings indicate marked local spatial heterogeneity at the provincial level, with both high-value and low-value provinces exhibiting clear clustering features and strong local spatial autocorrelation. Together with the Moran's *I* scatter plot, the results further suggest that the spatial evolution of the study object was not spatially balanced, but instead showed clear gradient differentiation and clustering effects across regions. The spatial distribution of cold spots and hot spots across 31 provinces in China in 2012, 2016, 2020, and 2023 is shown in Figure 3. Additional maps related to Figure 3 are provided in Supplementary Figures 1–12.

### 3.4 Gini coefficient analysis

**Figure 4:**
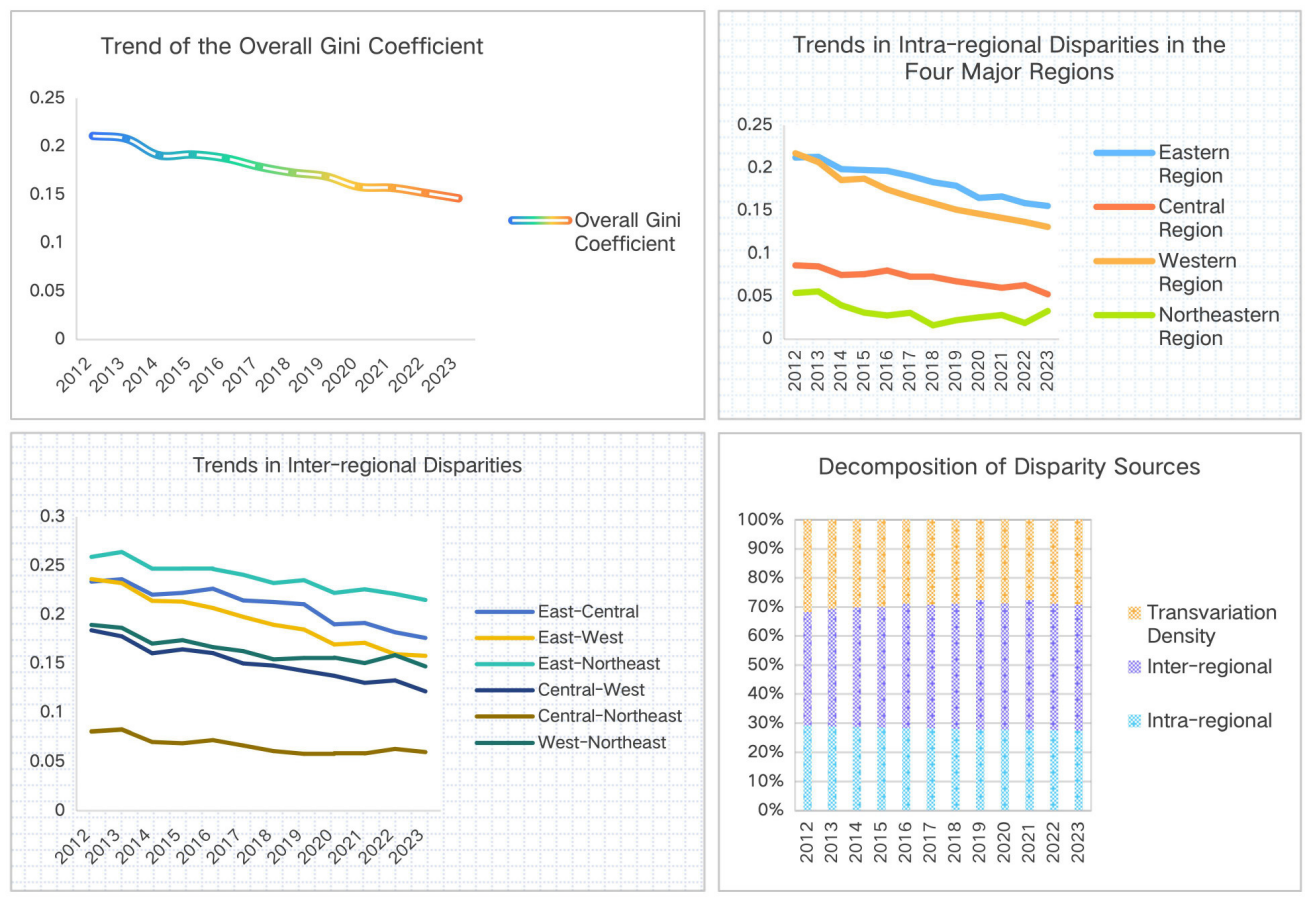
Trends in the Gini Coefficients of 31 Provinces in China, 2012–2023.

The overall Gini coefficient of the study object decreased from 0.2107 in 2012 to 0.1459 in 2023, with a mean of 0.1767, showing a continuous downward trend over the study period. This suggests an overall convergence in regional disparities during the sample period. Although the extent of regional inequality appeared to decline over time, the overall Gini coefficient remained at a certain level, indicating that regional disparities still persisted. The changing trend of the Gini coefficient across 31 provinces in China from 2012 to 2023 is shown in Figure 4.

With respect to intraregional differences, the mean within-region Gini coefficients for the eastern and western regions were 0.1844 and 0.1667, respectively, significantly higher than those for the central region (0.0711) and the northeastern region (0.0317). These results indicate that internal heterogeneity was more pronounced in the eastern and western regions, where intraregional development imbalance was relatively evident. Within-region disparity in the eastern region remained consistently high, possibly reflecting substantial differences among provinces in terms of economic foundations, industrial structure, and factor agglomeration capacity. The western region also showed marked internal disparities, likely associated with differences in resource endowment, infrastructure conditions, and stages of development across provinces. In contrast, the central and northeastern regions exhibited relatively small within-region differences, suggesting a more similar internal development pattern.

Interregional pairwise Gini coefficients generally declined over the study period, but between-region differences remained an important source of the overall disparity. The highest mean between-region Gini coefficient was observed between the eastern and northeastern regions (0.2380), followed by the eastern-central (0.2097) and eastern-western (0.1944) regional pairs, indicating that the development gap between the eastern region and the other regions was the most pronounced, especially relative to the northeastern region. By contrast, the lowest mean between-region Gini coefficient was found between the central and northeastern regions (0.0667), suggesting relatively small differences between these two regions. Overall, the eastern region exhibited a clear gradient pattern in relation to the other regions, making it an important contributor to overall regional disparity nationwide.

The decomposition results showed that, during the sample period, the mean contribution of interregional differences was 42.57%, exceeding that of intraregional differences (28.15%) and transvariation density (29.28%). This indicates that interregional differences were the largest contributor to the overall disparity. From 2012 to 2023, the contribution of interregional differences remained relatively stable at 39%−45% and consistently dominated the overall disparity. In contrast, the contribution of intraregional differences remained broadly stable at 27%−29%, and that of transvariation density showed only limited fluctuation. These results suggest some degree of overlap among regions, although its contribution was smaller than that of interregional differences.

Overall, although the overall Gini coefficient showed a downward trend during the study period, the underlying pattern of regional disparity did not fundamentally change. Interregional differences remained the dominant source of the overall disparity, with the gap between the eastern region and the other regions being particularly pronounced. Accordingly, subsequent policy efforts should place greater emphasis on cross-regional coordinated development by promoting the rational flow of production factors, strengthening regional cooperation, and optimizing the spatial layout of industries, thereby gradually alleviating interregional development imbalance and advancing higher-level regional coordinated development.

## 4 Discussion

This study yielded three principal findings. First, the composite score for traditional Chinese medicine service capacity showed an upward trend in all 31 provinces from 2012 to 2023, suggesting that the traditional Chinese medicine service system has expanded overall and continued to develop steadily. Second, comprehensive provincial capacity demonstrated significant and persistent positive spatial autocorrelation, with local clustering characterized by intensified hotspots in northern China and stable cold spots in southern China. Third, despite the continued convergence in overall national disparities, interregional differences remained the major source of overall inequality, and the gradient disparity between the eastern region and the other regions was still pronounced.

### 4.1 Overall improvement in service capacity and resource expansion

The results show that China's TCM service system expanded rapidly during 2012–2023. Growth was observed across all six indicators, suggesting that TCM has become an increasingly important part of the national healthcare system. This broad-based expansion is likely linked to the policy environment shaped by the Healthy China strategy, under which TCM has been more fully incorporated into national health planning and supported through institutional development, system reform, and public investment (9–11).

At the same time, the growth of institutions and bed capacity exceeded that of the traditional Chinese medicine workforce, which may indicate a structural coordination problem between resource expansion and human resource supply. Nevertheless, this inference should be interpreted with caution and further verified using institution-level data on workforce allocation and staff-to-post matching. This has implications for service quality, efficiency, and sustainability, especially in less-developed areas where workforce shortages are often more pronounced (12, 15, 22). In such contexts, increasing nominal capacity without adequate staffing may limit the actual performance of the service system.

The case of Xinjiang illustrates how coordinated planning can improve accessibility. By establishing a multilevel TCM service network spanning autonomous region, prefecture, county, township, and village levels, Xinjiang reportedly strengthened the reach of TCM services in primary care settings. This example suggests that expansion is likely to be more effective when institutional development is tied to explicit accessibility goals. However, because the present analysis is based on yearbook data, it cannot directly assess facility-level quality, staffing sufficiency, or service effectiveness.

Overall, these findings suggest that assessments of TCM development should move beyond simple measures of institutional growth and service volume. Greater attention should be paid to whether workforce development keeps pace with infrastructure expansion and whether human resources are distributed in a way that supports equitable access (4, 22).

### 4.2 Regional differentiation in TCM development

The study also shows that regional disparities in TCM service capacity remain substantial. According to the composite scores, Shanghai and Beijing consistently remained among the top-performing regions during the study period, underscoring the strong capacity of first-tier cities to concentrate resources and sustain service provision. Guangdong, Zhejiang, Jiangsu, and Shandong, all located in eastern China, also maintained comparatively high levels overall. At the same time, substantial improvements were observed in Sichuan, Chongqing, Inner Mongolia, Xinjiang, and Tibet, indicating that some provinces in central and western China experienced relatively rapid catch-up, likely driven by policy support and ongoing system development.

The persistent east–west divide likely reflects the combined influence of economic conditions, population concentration, transportation accessibility, and health resource agglomeration (5–8, 19). Eastern provinces appear to have benefited from stronger economic foundations, denser populations, better transport links, and a concentration of high-level institutions, all of which may have facilitated the strengthening of TCM service capacity. Western provinces, by contrast, face structural constraints associated with dispersed settlement patterns, difficult terrain, lower population density, and longer travel distances. Under such conditions, institutional expansion may not necessarily translate into equivalent gains in accessibility or utilization (16, 17).

Central China exhibited a somewhat different pattern. Provinces such as Henan, Hunan, and Hubei showed rapid growth, suggesting that healthcare reform and strong service demand in densely populated areas may have accelerated the translation of policy inputs into measurable improvements. This may help explain the relative convergence observed in the central region.

Intraregional disparities displayed a further layer of complexity. With respect to intraregional variation, Table 5 indicates that the mean Gini coefficients within eastern China and western China were 0.1844 and 0.1667, respectively, exceeding those observed in central China (0.0711) and northeastern China (0.0317). These results imply stronger internal heterogeneity in the eastern and western regions, while the central and northeastern regions exhibited relatively smaller internal disparities. The mechanisms underlying this pattern are likely to differ across regions. In eastern China, internal inequality may be associated with uneven concentration of high-quality resources and developmental gradients among provinces. In western China, it may be more strongly driven by geographic barriers, long travel distances, and limited accessibility (16, 17). Although both regions exhibited relatively high internal inequality, the structural sources of that inequality appear to be distinct.

**Table 5:** Gini coefficients of 31 provinces in China, 2012–2023.

year	Overall	Intra-group Disparity			Inter-group Disparity			Contribution to Disparity (%)						
		Eastern region	Central region	Western region	Northeastern region	East-Central	East-West	East-Northeast	Central-West	Central-Northeast	West-Northeast	Intra-regional	Inter-regional	Transvariation density
2012	0.2107	0.2118	0.0861	0.2168	0.0538	0.2337	0.2363	0.2590	18.40%	8.08%	18.95%	29.14%	39.11%	31.75%
2013	0.2078	0.2127	0.0849	0.2065	0.0556	0.2362	0.2322	0.2640	17.78%	8.28%	18.65%	28.88%	40.51%	30.61%
2014	0.1905	0.1983	0.0749	0.1855	0.0394	0.2204	0.2141	0.2470	16.05%	7.02%	17.04%	28.62%	41.26%	30.12%
2015	0.1914	0.1972	0.0758	0.1871	0.0308	0.2222	0.2133	0.2471	16.45%	6.89%	17.39%	28.55%	41.50%	29.95%
2016	0.1874	0.1963	0.0801	0.1745	0.0275	0.2265	0.2068	0.2466	16.07%	7.21%	16.68%	28.21%	42.93%	28.85%
2017	0.1788	0.1905	0.0729	0.1659	0.0306	0.2145	0.1977	0.2407	15.01%	6.66%	16.27%	28.27%	42.50%	29.23%
2018	0.1727	0.1830	0.0726	0.1585	0.0160	0.2129	0.1896	0.2323	14.79%	6.09%	15.43%	28.02%	43.11%	28.86%
2019	0.1688	0.1789	0.0675	0.1509	0.0219	0.2106	0.1848	0.2352	14.25%	5.81%	15.57%	27.65%	44.74%	27.61%
2020	0.1579	0.1646	0.0637	0.1460	0.0253	0.1901	0.1695	0.2222	13.75%	5.87%	15.61%	27.85%	43.36%	28.80%
2021	0.1568	0.1663	0.0599	0.1413	0.0280	0.1915	0.1712	0.2259	13.03%	5.85%	15.05%	27.63%	44.79%	27.58%
2022	0.1515	0.1585	0.0630	0.1365	0.0187	0.1820	0.1596	0.2212	13.28%	6.31%	15.86%	27.55%	43.62%	28.83%
2023	0.1459	0.1552	0.0523	0.1307	0.0327	0.1762	0.1579	0.2150	12.16%	5.99%	14.71%	27.47%	43.35%	29.18%
Average	0.1767	0.1844	0.0711	0.1667	0.0317	0.2097	0.1944	0.2380	0.1509	0.0667	0.1643	0.2815	0.4257	0.2928

It is also noteworthy that intraregional inequality increased in both eastern and western China after 2020. One possible explanation is that public health emergencies, including the COVID-19 pandemic, amplified pre-existing asymmetries in fiscal capacity and health system resilience (23, 24). Provinces with stronger institutional and financial bases may have been better able to maintain or adapt TCM services during periods of disruption, whereas less-resourced areas may have faced greater constraints.

### 4.3 Dynamic equity and overlapping disparities

The decomposition results reveal a more nuanced pattern of inequality than can be captured by aggregate regional comparisons alone. The overall Gini coefficient for traditional Chinese medicine service capacity showed a tendency toward convergence between 2012 and 2023. Nevertheless, the decomposition results indicated that interregional differences contributed more to overall inequality than intraregional differences and transvariation density, implying that disparities in traditional Chinese medicine service capacity across China were still mainly driven by development gradients between regions. In particular, the mean interregional differences between eastern China and the other regions remained relatively large, highlighting the persistent comparative advantage of eastern China. At the same time, the average contribution of transvariation density remained broadly stable throughout the study period, suggesting that provincial development levels within regions were not strictly aligned with conventional geographic groupings, but instead showed notable cross-regional overlap. This finding is consistent with the local clustering patterns and cross-regional heterogeneity identified in the spatial analysis, and it further indicates that policy formulation should move beyond broad regional categorizations and give greater consideration to province-level heterogeneity and differentiated governance.

A key finding is the persistently large contribution of transvariation density to overall inequality. This indicates that the distribution of TCM service capacity cannot be fully explained by a simple distinction between within-region and between-region differences. Instead, there is substantial overlap across regions, with some provinces in less-developed areas outperforming provinces in more-developed regions, and some provinces in economically stronger areas still lagging behind. This suggests that conventional regional categories are useful but insufficient for fully capturing the actual geography of TCM development (9).

The prominence of transvariation density may also reflect the coexistence of resource concentration and resource scarcity. In some provinces, TCM resources may be highly concentrated, reinforcing local advantages in accessibility and utilization. In others, shortages of institutions, personnel, or service capacity may continue to limit the development of basic services. When these contrasting patterns coexist across regions, the resulting distribution becomes more heterogeneous and overlapping, thereby increasing overall inequality.

From a public health perspective, this finding points to the limitations of relying exclusively on broad regional categories when designing allocation policies. Although narrowing average regional gaps remains important, greater attention should also be paid to cross-regional stratification and province-level heterogeneity. A more fine-grained approach is therefore needed to improve the fairness and effectiveness of TCM resource allocation (1, 4, 17).

## 5 Policy implications

The findings suggest that, despite the overall improvement in traditional Chinese medicine service capacity, interprovincial disparities persist, accompanied by stable spatial clustering and pronounced regional gradients. Therefore, policy efforts should extend beyond the expansion of aggregate resources and shift toward a more comprehensive governance approach that emphasizes cross-regional coordination, targeted allocation, and spatially balanced development.

First, workforce development should remain a central priority. Given the potential mismatch between infrastructure growth and personnel allocation, stronger efforts are needed to improve the training, recruitment, retention, and continuing education of TCM professionals. Particular attention should be given to shortage-related specialties and to primary healthcare settings in central, western, and rural areas, where staffing constraints may be most severe (22). More flexible staffing adjustment mechanisms may also help align human resources with service demand and institutional performance.

Second, regional coordination should be further strengthened. As interregional differences accounted for a mean contribution of 42.57%, markedly higher than that of intraregional differences and transvariation density, policy interventions should primarily focus on reducing regional development gradients. Priority should be given to narrowing resource gaps between eastern China and the central, western, and northeastern regions through the establishment of more sustainable cross-regional cooperation mechanisms, such as paired support from high-level traditional Chinese medicine hospitals, flexible deployment of health personnel, telemedicine-based collaboration, and specialty alliance development. In central, western, and remote areas, fiscal support should be further directed toward the construction of primary traditional Chinese medicine institutions, equipment upgrading, and workforce retention so as to strengthen comprehensive service capacity. Meanwhile, within provinces, particularly in eastern and western China, stronger measures are needed to facilitate the redistribution of high-quality resources to the primary level and to reinforce cooperation between top-tier traditional Chinese medicine hospitals and grassroots healthcare institutions (5, 16).

Third, beyond public-sector investment and interregional assistance, further improvement in the equity of TCM services may also require greater attention to diversified provision mechanisms, digital health development, and insurance-based incentives (17, 25). Policy guidance could be used to encourage private-sector participation in resource-deficient areas, thereby complementing public-sector provision and expanding service availability in underserved regions. Surplus resources from some urban TCM hospitals could be redirected toward primary healthcare institutions and suburban areas to promote a more balanced spatial distribution of services. In planning the TCM service system, newly added institutional capacity should be preferentially allocated to areas with service gaps or limited service capacity, so that incremental expansion is better aligned with unmet population needs.

Fourth, the consistently positive and statistically significant global Moran's *I* from 2012 to 2023, together with the local pattern of “intensified northern hotspots and stable southern cold spots,” indicates the need for policy interventions that specifically address spatial deficiencies in cold-spot areas. Considering the coexistence of regional disparities and spatial clustering, a stratified and region-specific policy framework may be more appropriate. Eastern China should place greater emphasis on the internal redistribution of high-quality resources so as to mitigate the excessive concentration of advanced services across provinces and between urban and rural areas. Central China should further consolidate its relative convergence trend and continue to improve service capacity and conversion efficiency in densely populated provinces. Western and frontier regions should prioritize the development of primary traditional Chinese medicine institutions, workforce retention, and cross-regional referral coordination to improve service accessibility. Northeastern China, while maintaining its relatively balanced development, should focus more on enhancing service quality and health system resilience. In persistent cold-spot provinces, including Guangxi, Hainan, and Guizhou, digital infrastructure, primary-level workforce support, and paired assistance resources should be prioritized to break the lock-in effect of low-value clustering (17, 25). Expanding health insurance coverage for TCM services, together with exploring payment methods that better reflect the characteristics of TCM, may also strengthen incentives for efficient and high-quality service delivery.

These policy directions are especially important at a stage when TCM development is shifting from quantitative expansion toward quality-oriented improvement. In this context, narrowing disparities will depend not only on increasing the scale of resources, but also on continued institutional and policy innovation aimed at improving equity in TCM service capacity and promoting a more equitable distribution of TCM resources. Meanwhile, the high-quality development of traditional Chinese medicine increasingly depends on the coordination between the innovation chain and the industrial chain, digital empowerment, and the cultivation of new quality productive forces (26), which together provide broader external support for improving the modernization of the TCM service system.

## 6 Research contributions

Compared with previous studies, this study makes three main contributions. First, in terms of indicator design, it not only evaluates the scale of TCM service supply and utilization, but also incorporates regional comparative variables, including GDP per capita, resident population, administrative area, and population density, thereby improving the comparability of the provincial-level composite assessment. Second, in terms of methodology, it integrates entropy-weighted composite evaluation, global and local spatial autocorrelation analysis based on an economic-geographic weight matrix, and Dagum Gini coefficient decomposition, thus establishing an integrated analytical framework of “composite capacity identification-spatial pattern characterization-source decomposition of regional disparities.” Third, in terms of study scope, by examining the long-term evolution of TCM service capacity across 31 provinces in China from 2012 to 2023, this study identifies two parallel features: the strengthening of northern hot spots alongside the persistence of southern cold spots, and an overall convergence trend accompanied by regional disparities that remain primarily driven by interregional differences. These findings provide more targeted empirical evidence for the spatial governance and optimized allocation of TCM resources.

## 7 Limitations

This study contributes to the existing literature in three main ways. First, it extends indicator design beyond the scale of traditional Chinese medicine service supply and utilization by incorporating regional comparative variables, such as per capita GDP, resident population, administrative area, and population density, thus enhancing the cross-provincial comparability of the comprehensive assessment. Second, it develops an integrated analytical framework that combines entropy-weighted comprehensive evaluation, global and local spatial autocorrelation analysis under an economic-geographic weight matrix, and Dagum Gini coefficient decomposition, enabling the identification of overall capacity, the characterization of spatial patterns, and the decomposition of disparity sources. Third, although the spatial correlation analysis identified significant clustering patterns, it did not further examine the specific mechanisms through which economic, demographic, transportation, and policy-related factors shape these patterns by using spatial econometric models. Previous studies have explored methodological approaches from the perspective of efficiency and its determinants, including DEA and configurational analysis (15); however, the mechanisms underlying spatial disparities in TCM service capacity remain insufficiently investigated.

Fourth, the small number of missing values was imputed using mean substitution, which may have underestimated the actual degree of dispersion across certain years and regions. Fifth, this study is primarily descriptive and decomposition-based and therefore cannot identify the causal effects of specific policy interventions. Future research could build on institution-level data and apply spatial panel models and quasi-experimental designs to provide more robust evidence on these issues (16, 17, 27).

It should also be acknowledged that the interpretation of factors such as economic development, population concentration, transport conditions, and policy support is mainly derived from existing literature and the observed characteristics of the results, rather than from formal causal inference. As this study primarily focuses on identifying spatiotemporal disparities in traditional Chinese medicine service capacity and decomposing the sources of inequality, the related driving mechanisms remain to be further tested in future studies using methods such as spatial panel regression, threshold models, or mediation-effect models.

## 8 Conclusion

Between 2012 and 2023, traditional Chinese medicine service capacity in 31 provinces of China improved continuously, reflecting rapid development in resource supply, service utilization, and workforce deployment. However, despite an overall narrowing of interprovincial disparities, such inequalities persisted. Although the overall Dagum Gini coefficient declined, interregional differences continued to account for the largest share of overall inequality, with a clear gradient between eastern China and the other regions. In spatial terms, significant positive spatial autocorrelation was consistently identified throughout the study period, while the local pattern of “strengthening northern hotspots and stable southern cold spots” remained evident. These findings indicate that traditional Chinese medicine service capacity is characterized by marked spatial clustering and regional interdependence. Accordingly, future policy should not only continue to expand service capacity, but also strengthen cross-regional coordination, prioritize gap-filling in persistent cold-spot areas, promote the targeted downward redistribution of talent and resources, and enhance digital support, thereby fostering more equitable and sustainable development.

These findings suggest that progress in TCM development should not be judged solely by aggregate expansion. Greater emphasis should be placed on spatial equity, coordinated workforce development, and differentiated policy design. Achieving more balanced and sustainable development of TCM services in China will require not only continued resource growth, but also more precise institutional planning and policy innovation tailored to enduring regional and cross-regional disparities.

## Data Availability

The original contributions presented in the study are included in the article/Supplementary material, further inquiries can be directed to the corresponding author.

## References

[1] World Health Organization. The World Health Report 2000: Health Systems: Improving Performance. Geneva: World Health Organization(2000).

[2] Whitehead M. The concepts and principles of equity and health. Int J Health Serv. (1992) 22:429–45. doi: 10.2190/986L-LHQ6-2VTE-YRRN 1644507

[3] Braveman P Gruskin S. Defining equity in health. J Epidemiol Community Health. (2003) 57:254–8. doi: 10.1136/jech.57.4.254 12646539 PMC1732430

[4] Starfield B. State of the art in research on equity in health. J Health Polit Policy Law. (2006) 31:11–32. doi: 10.1215/03616878-31-1-11 16484666

[5] Anand S Fan VY Zhang J Zhang L Ke Y Dong Z . China's human resources for health: quantity, quality, and distribution. Lancet. (2008) 372:1774–81. doi: 10.1016/S0140-6736(08)61363-X 18930528

[6] Yip W Hsiao WC Chen W Hu S Ma J Maynard A. Early appraisal of China's huge and complex health-care reforms. Lancet. (2012) 379:833–42. doi: 10.1016/S0140-6736(11)61880-1 22386036

[7] Li X Krumholz HM Yip W Cheng KK De Maeseneer J Meng Q . Quality of primary health care in China: challenges and recommendations. Lancet. (2020) 395:1802–12. doi: 10.1016/S0140-6736(20)30122-7 32505251 PMC7272159

[8] Meng Q Mills A Wang L Han Q. What can we learn from China's health system reform? BMJ. (2019) 365:l2349. doi: 10.1136/bmj.l2349 31217222 PMC6598719

[9] World Health Organization. WHO Traditional Medicine Strategy: 2014–2023. Geneva: World Health Organization (2013).

[10] National Administration of Traditional Chinese Medicine. Statistical Report on the Development of Traditional Chinese Medicine in China. Available online at: http://www.natcm.gov.cn/guicaisi/gongzuodongtai/2022-01-20/24293.html (Accessed 18 May, 2026).

[11] The State Council of the People's Republic of China. Outline of the Healthy China 2030 Plan. Available online at: http://www.gov.cn/zhengce/2016-10/25/content_5124174.htm (Accessed 18 May, 2026).

[12] Zhang X Zhao L Cui Z Wang Y. Study on equity and efficiency of health resources and services based on key indicators in China. PLoS ONE. (2015) 10:e0144809. doi: 10.1371/journal.pone.0144809 26679187 PMC4683010

[13] McGrail MR Humphreys JS. Measuring spatial accessibility to primary care in rural areas: improving the effectiveness of the two-step floating catchment area method. Appl Geogr. (2009) 29:533–41. doi: 10.1016/j.apgeog.2008.12.003

[14] Li ZG Wei H. A comprehensive evaluation of China's TCM medical service system: an empirical research by integrated factor analysis and TOPSIS. Front Public Health. (2020) 8:532420. doi: 10.3389/fpubh.2020.532420 33117767 PMC7550738

[15] Li Z Zhang W Kong A Ding Z Wei H Guo Y. Configuration analysis of influencing factors of technical efficiency based on DEA and fsQCA: evidence from China's medical and health institutions. Risk Manag Healthc Policy. (2021) 49–65. doi: 10.2147/RMHP.S282178 33447109 PMC7802899

[16] Guagliardo MF. Spatial accessibility of primary care: concepts, methods and challenges. Int J Health Geogr. (2004) 3:3. doi: 10.1186/1476-072X-3-3 14987337 PMC394340

[17] Wang F. Measurement, optimization, and impact of health care accessibility: a methodological review. Ann Assoc Am Geogr. (2012) 102:1104–12. doi: 10.1080/00045608.2012.657146 23335813 PMC3547595

[18] Delamater PL Messina JP Shortridge AM Grady SC. Measuring geographic access to health care: raster and network-based methods. Int J Health Geogr. (2012) 11:15. doi: 10.1186/1476-072X-11-15 22587023 PMC3511293

[19] Wang Z Li Z Xie R. Regional disparities, dynamic evolution, and spatial spillover effects of medical resource allocation efficiency in TCM hospitals. Cost Eff Resour Alloc. (2025) 23:35. doi: 10.1186/s12962-025-00644-6 40682090 PMC12273394

[20] Dagum C. A new approach to the decomposition of the Gini income inequality ratio. Empir Econ. (1997) 22:515–31. doi: 10.1007/BF01205777

[21] Shannon CE. A mathematical theory of communication. Bell Syst Tech J. (1948) 27:379–423. doi: 10.1002/j.1538-7305.1948.tb01338.x

[22] Chen Z. Launch of the health-care reform plan in China. Lancet. (2009) 373:1322–4. doi: 10.1016/S0140-6736(09)60753-4 19376436

[23] Bambra C Riordan R Ford J Matthews F. The COVID-19 pandemic and health inequalities. J Epidemiol Community Health. (2020) 74:964–8. doi: 10.1136/jech-2020-214401 32535550 PMC7298201

[24] Ahmed F Ahmed N Pissarides C Stiglitz J. Why inequality could spread COVID-19. Lancet Public Health. (2020) 5:e240. doi: 10.1016/S2468-2667(20)30085-2 32247329 PMC7270465

[25] Keesara S Jonas A Schulman K. Covid-19 and health care's digital revolution. N Engl J Med. (2020) 382:e82. doi: 10.1056/NEJMp2005835 32240581

[26] Li Z Zhao W Zhang F Xie R. Investigating the impact of dual-chain coupling on new quality productive forces in leading traditional Chinese medicine enterprises. Front Med. (2026) 13:1828456. doi: 10.3389/fmed.2026.1828456 PMC13243264 42266963

[27] Diez Roux AV. Investigating neighborhood and area effects on health. Am J Public Health. (2001) 91:1783–9. doi: 10.2105/AJPH.91.11.1783 11684601 PMC1446876

