# Supplementary material for: Multi-index spatio-temporal joint evaluation of traditional Chinese medicine service capacity: empirical analysis of china provincial panel data from 2012 to 2023
Source: Front Public Health. 2026 Jul 28;14:1884758. doi: 10.3389/fpubh.2026.1884758 (PMC13457043; doi:10.3389/fpubh.2026.1884758)
Supplement: Supplementary file 1 [file Presentation_1.pdf]

Supplementary Material

This supplementary file provides additional annual hotspot-coldspot maps related to Figure 3 in the main text. Using the Getis-Ord Gi* statistic based on the economic-geographic weight matrix, Supplementary Figures S1-S12 present the yearly local spatial clustering patterns of traditional Chinese medicine (TCM) service capacity across 31 provincial-level regions in China from 2012 to 2023. These figures are intended to provide a more detailed year-by-year visualization of the spatial evolution of high-value and low-value clusters described in the manuscript.

Figure 3 in the main text presents selected representative years (2012, 2016, 2020, and 2023), whereas the present supplementary file provides the full year-by-year maps for the entire study period from 2012 to 2023.

2012

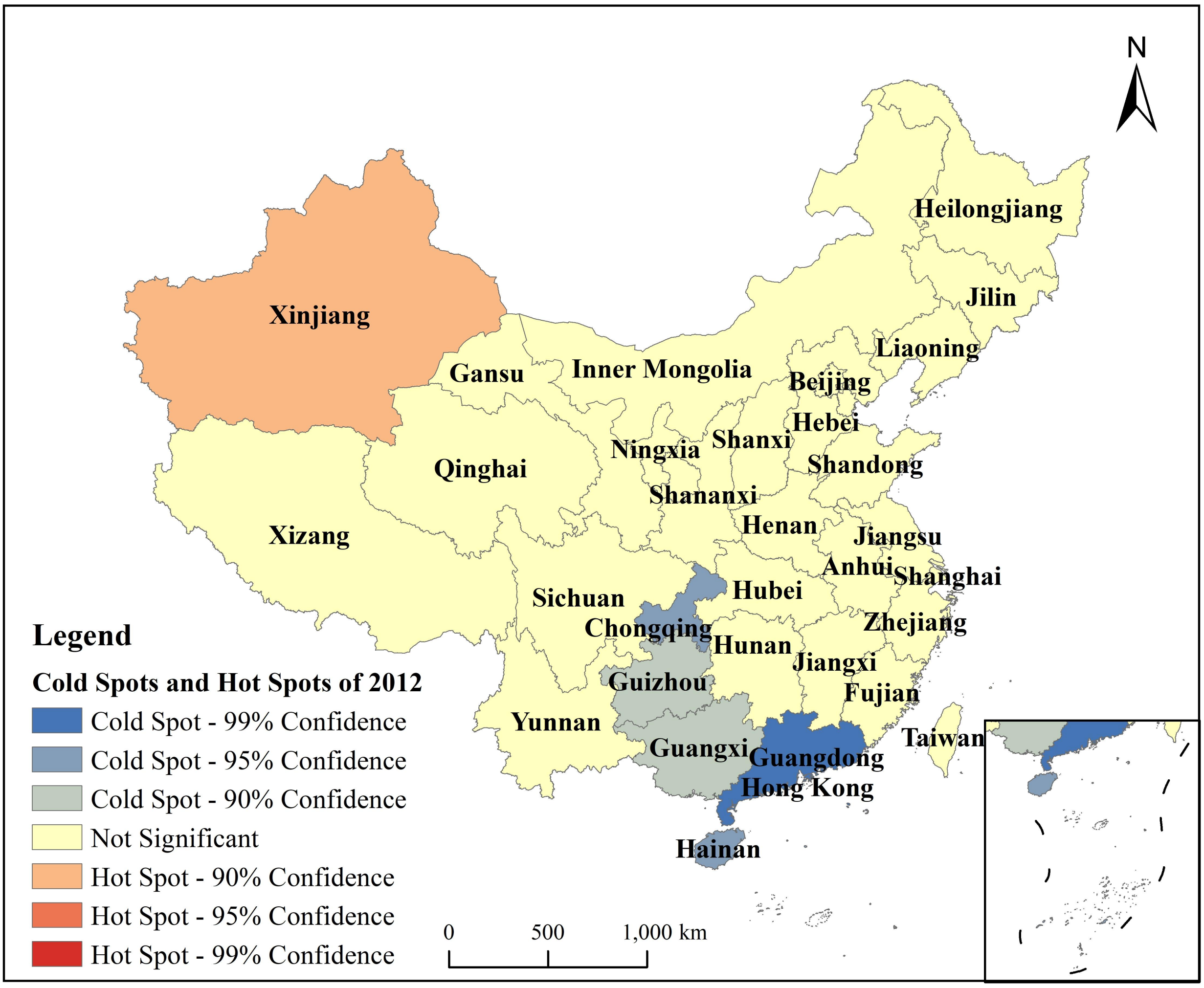

Supplementary Figure S1. Annual hotspot-coldspot map of traditional Chinese medicine (TCM) service capacity across 31 provincial-level regions in China in 2012. Local spatial clustering was identified using the Getis-Ord Gi* statistic under the economic-geographic weight matrix. Red shades indicate hot spots (high-value clusters), blue shades indicate cold spots (low-value clusters), and darker colors represent higher levels of statistical significance.

2013

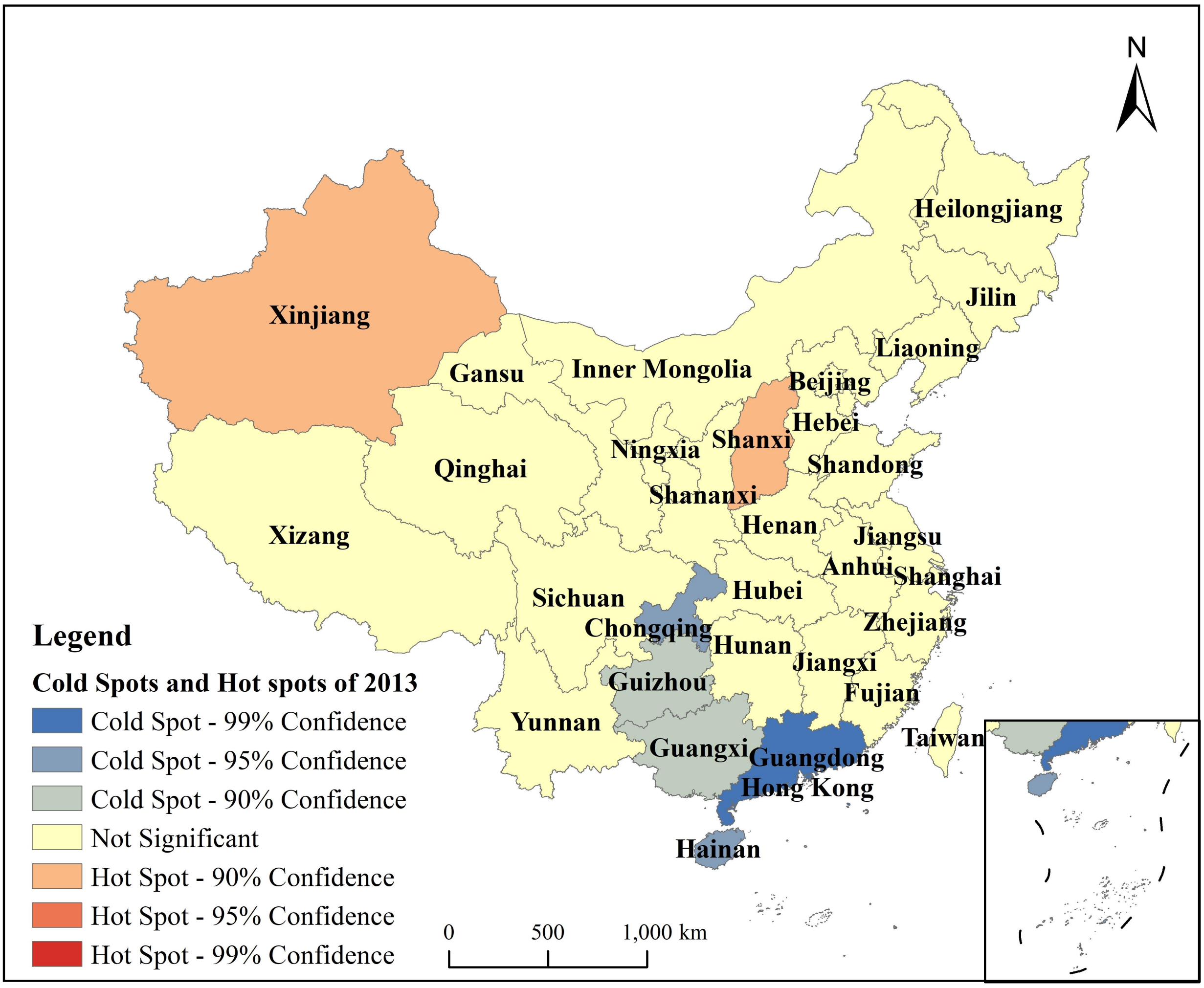

Supplementary Figure S2. Annual hotspot-coldspot map of traditional Chinese medicine (TCM) service capacity across 31 provincial-level regions in China in 2013. Local spatial clustering was identified using the Getis-Ord Gi* statistic under the economic-geographic weight matrix. Red shades indicate hot spots (high-value clusters), blue shades indicate cold spots (low-value clusters), and darker colors represent higher levels of statistical significance.

2014

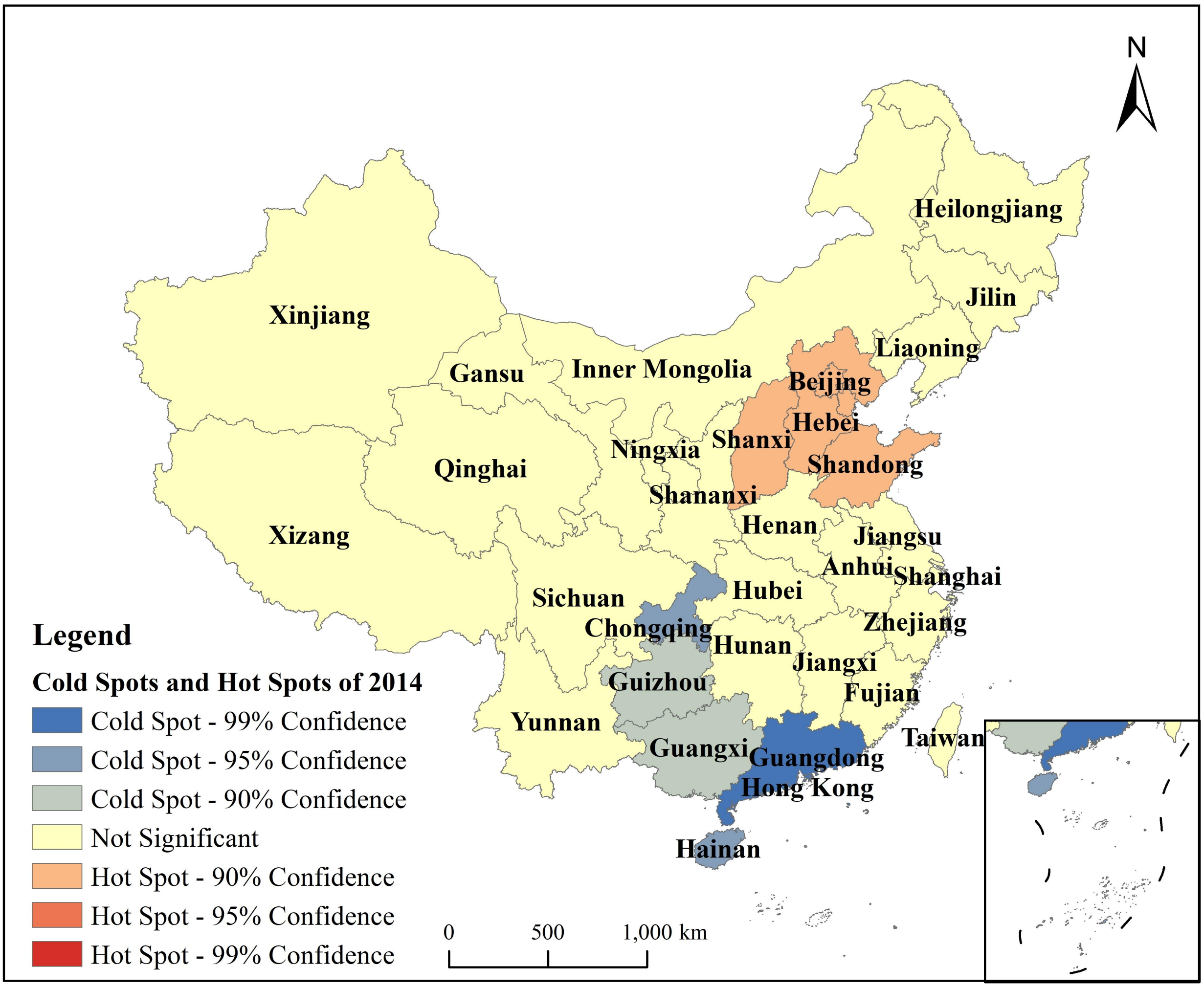

Supplementary Figure S3. Annual hotspot-coldspot map of traditional Chinese medicine (TCM) service capacity across 31 provincial-level regions in China in 2014. Local spatial clustering was identified using the Getis-Ord Gi* statistic under the economic-geographic weight matrix. Red shades indicate hot spots (high-value clusters), blue shades indicate cold spots (low-value clusters), and darker colors represent higher levels of statistical significance.

2015

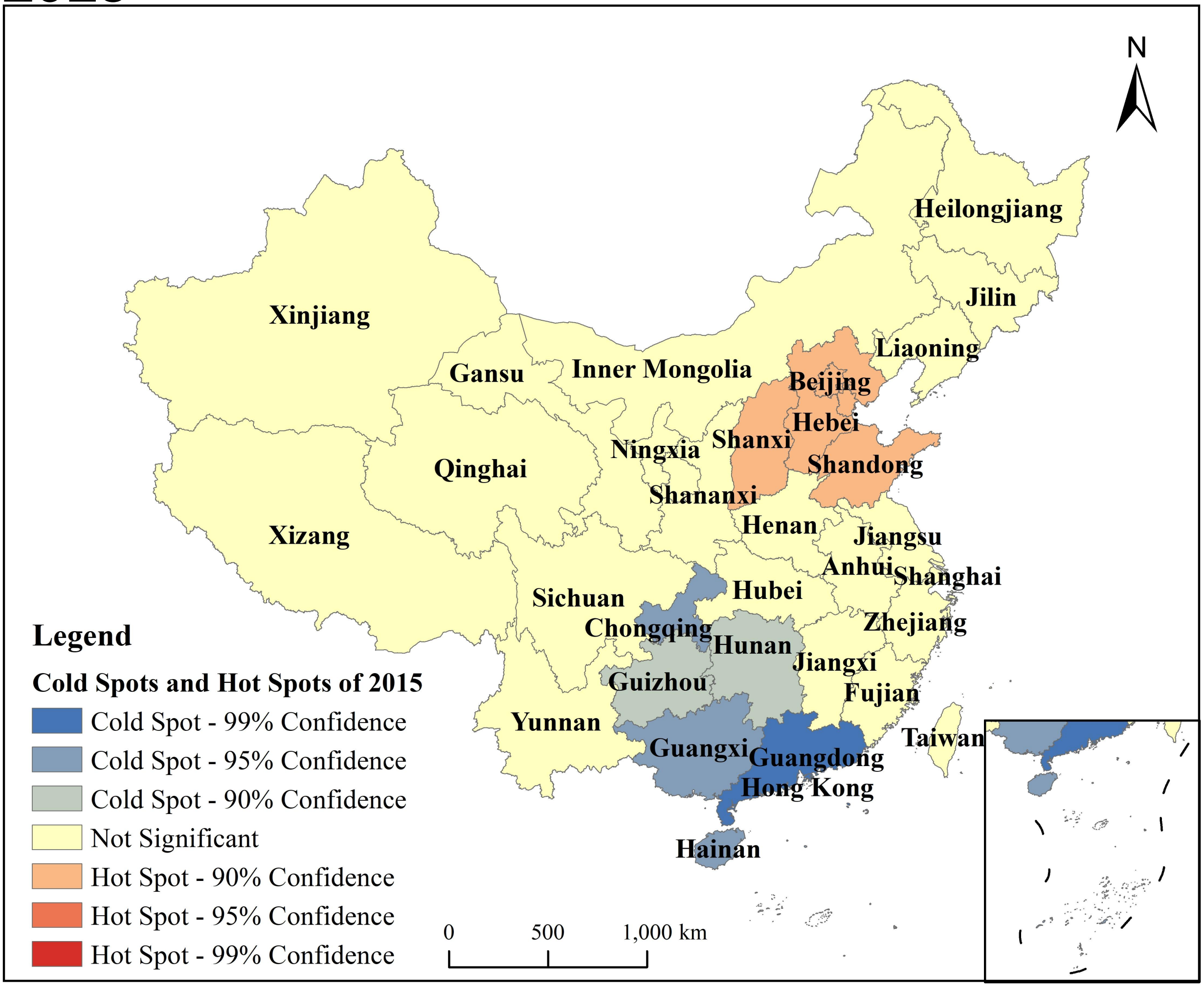

Supplementary Figure S4. Annual hotspot-coldspot map of traditional Chinese medicine (TCM) service capacity across 31 provincial-level regions in China in 2015. Local spatial clustering was identified using the Getis-Ord Gi* statistic under the economic-geographic weight matrix. Red shades indicate hot spots (high-value clusters), blue shades indicate cold spots (low-value clusters), and darker colors represent higher levels of statistical significance.

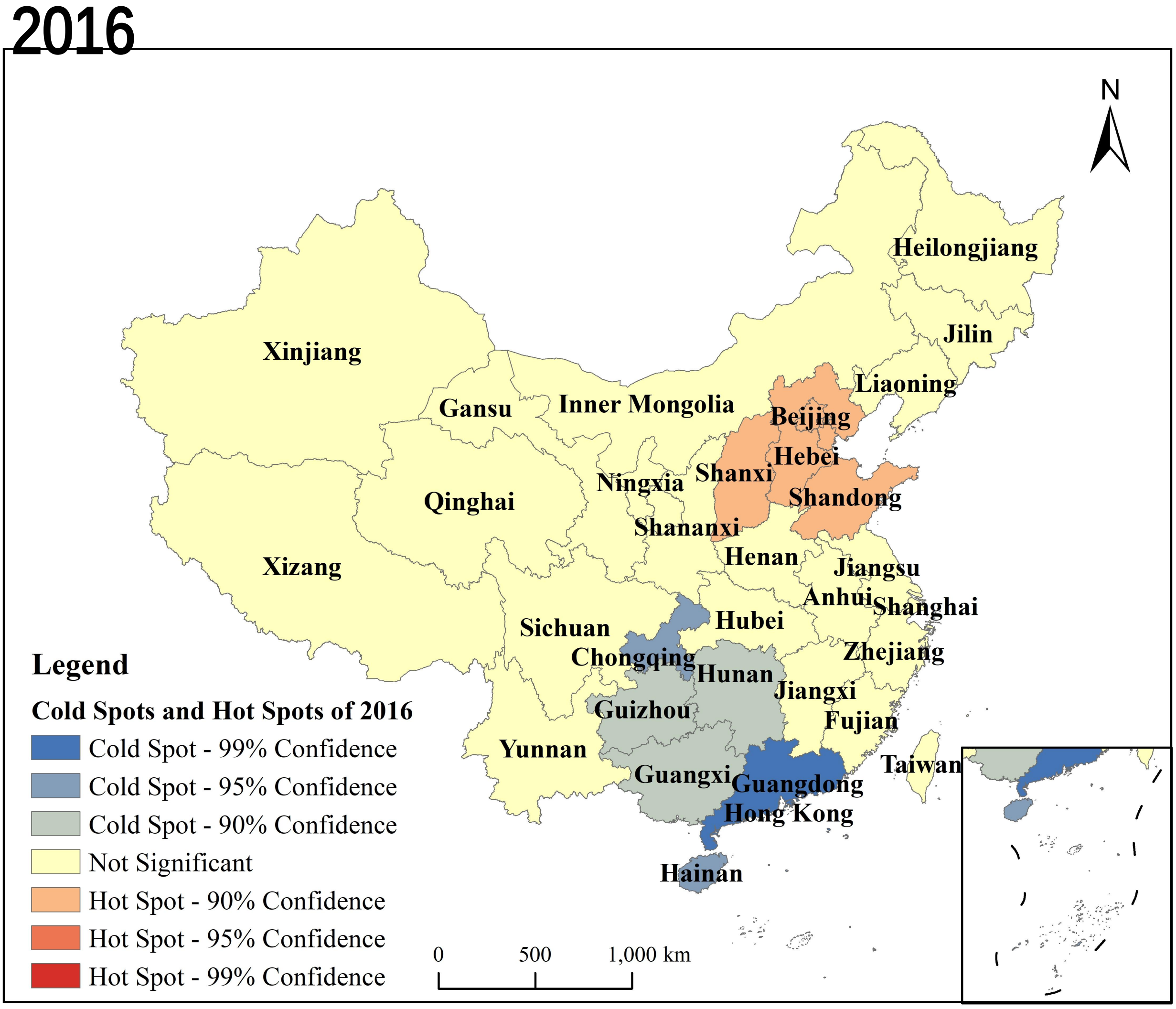

Supplementary Figure S5. Annual hotspot-coldspot map of traditional Chinese medicine (TCM) service capacity across 31 provincial-level regions in China in 2016. Local spatial clustering was identified using the Getis-Ord Gi* statistic under the economic-geographic weight matrix. Red shades indicate hot spots (high-value clusters), blue shades indicate cold spots (low-value clusters), and darker colors represent higher levels of statistical significance.

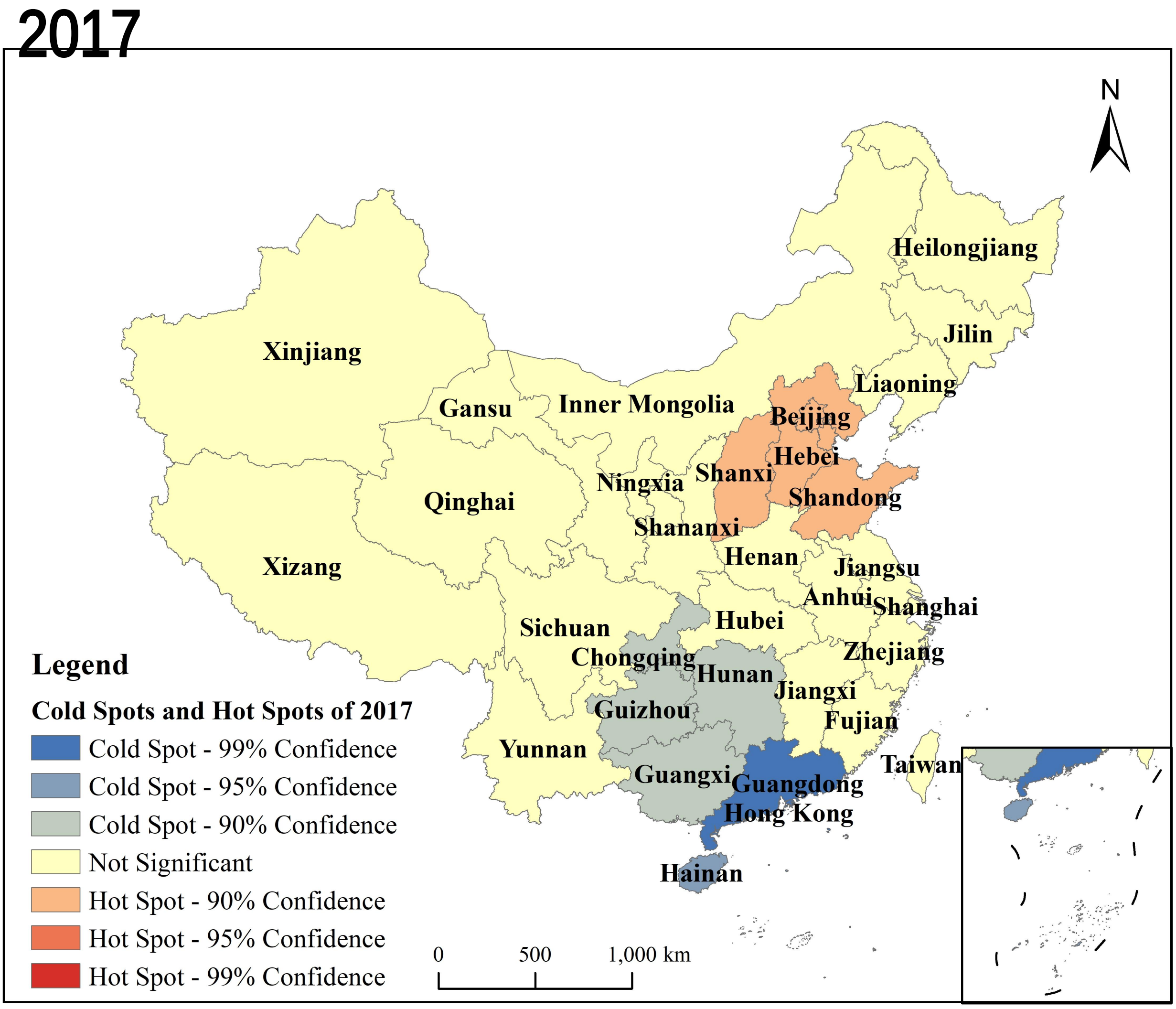

Supplementary Figure S6. Annual hotspot-coldspot map of traditional Chinese medicine (TCM) service capacity across 31 provincial-level regions in China in 2017. Local spatial clustering was identified using the Getis-Ord Gi* statistic under the economic-geographic weight matrix. Red shades indicate hot spots (high-value clusters), blue shades indicate cold spots (low-value clusters), and darker colors represent higher levels of statistical significance.

2018

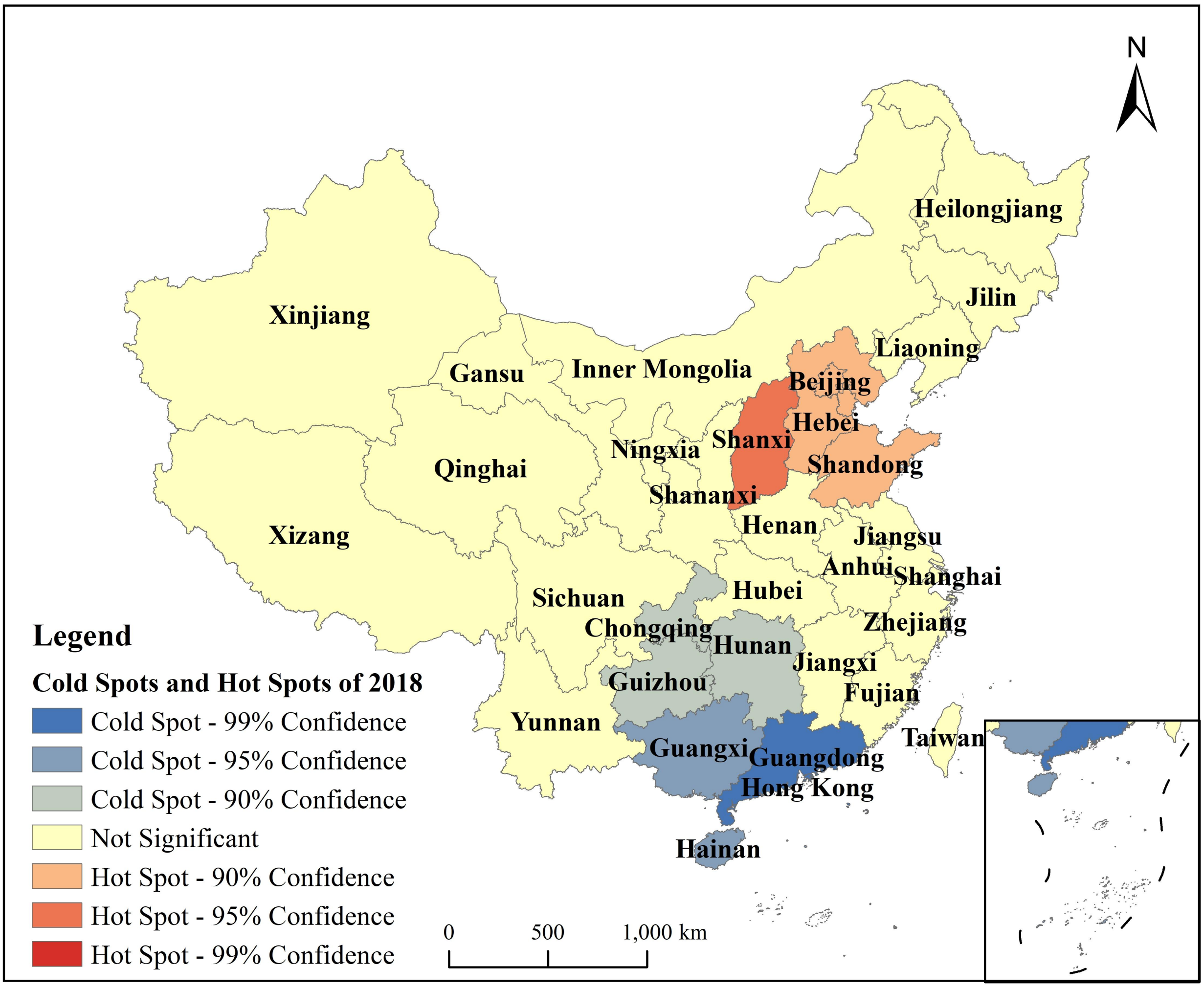

Supplementary Figure S7. Annual hotspot-coldspot map of traditional Chinese medicine (TCM) service capacity across 31 provincial-level regions in China in 2018. Local spatial clustering was identified using the Getis-Ord Gi* statistic under the economic-geographic weight matrix. Red shades indicate hot spots (high-value clusters), blue shades indicate cold spots (low-value clusters), and darker colors represent higher levels of statistical significance.

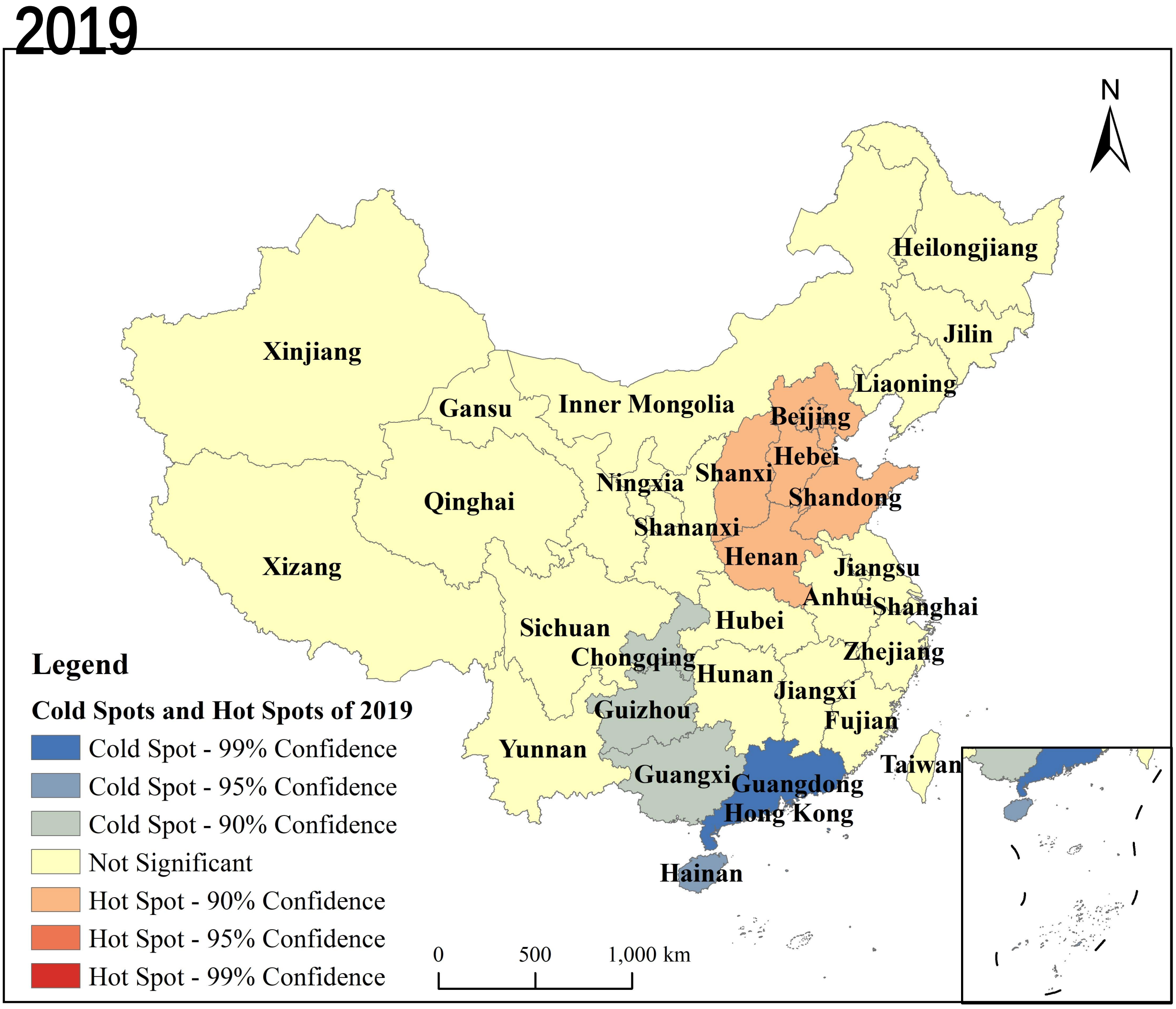

Supplementary Figure S8. Annual hotspot-coldspot map of traditional Chinese medicine (TCM) service capacity across 31 provincial-level regions in China in 2019. Local spatial clustering was identified using the Getis-Ord Gi* statistic under the economic-geographic weight matrix. Red shades indicate hot spots (high-value clusters), blue shades indicate cold spots (low-value clusters), and darker colors represent higher levels of statistical significance.

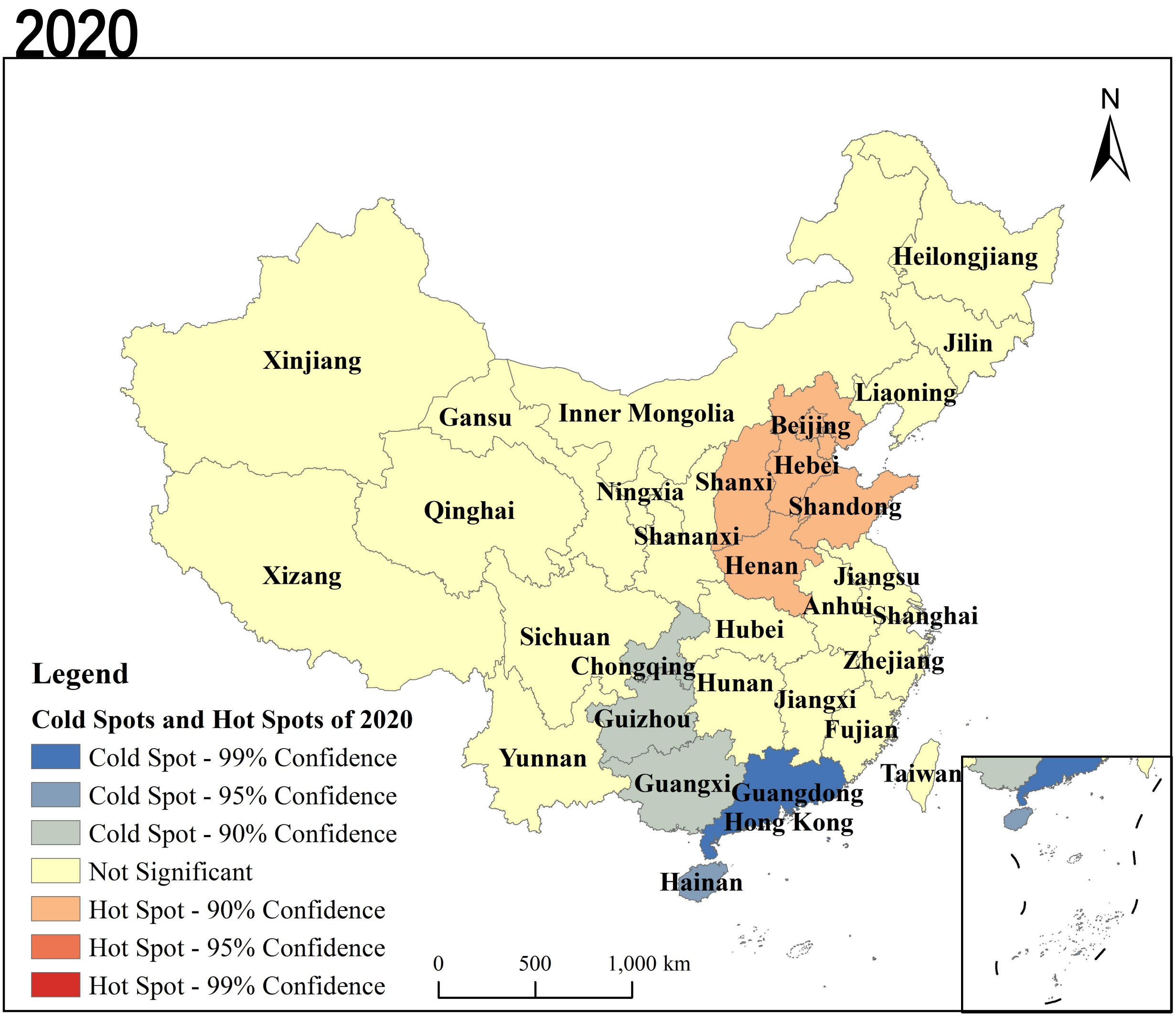

Supplementary Figure S9. Annual hotspot-coldspot map of traditional Chinese medicine (TCM) service capacity across 31 provincial-level regions in China in 2020. Local spatial clustering was identified using the Getis-Ord Gi* statistic under the economic-geographic weight matrix. Red shades indicate hot spots (high-value clusters), blue shades indicate cold spots (low-value clusters), and darker colors represent higher levels of statistical significance.

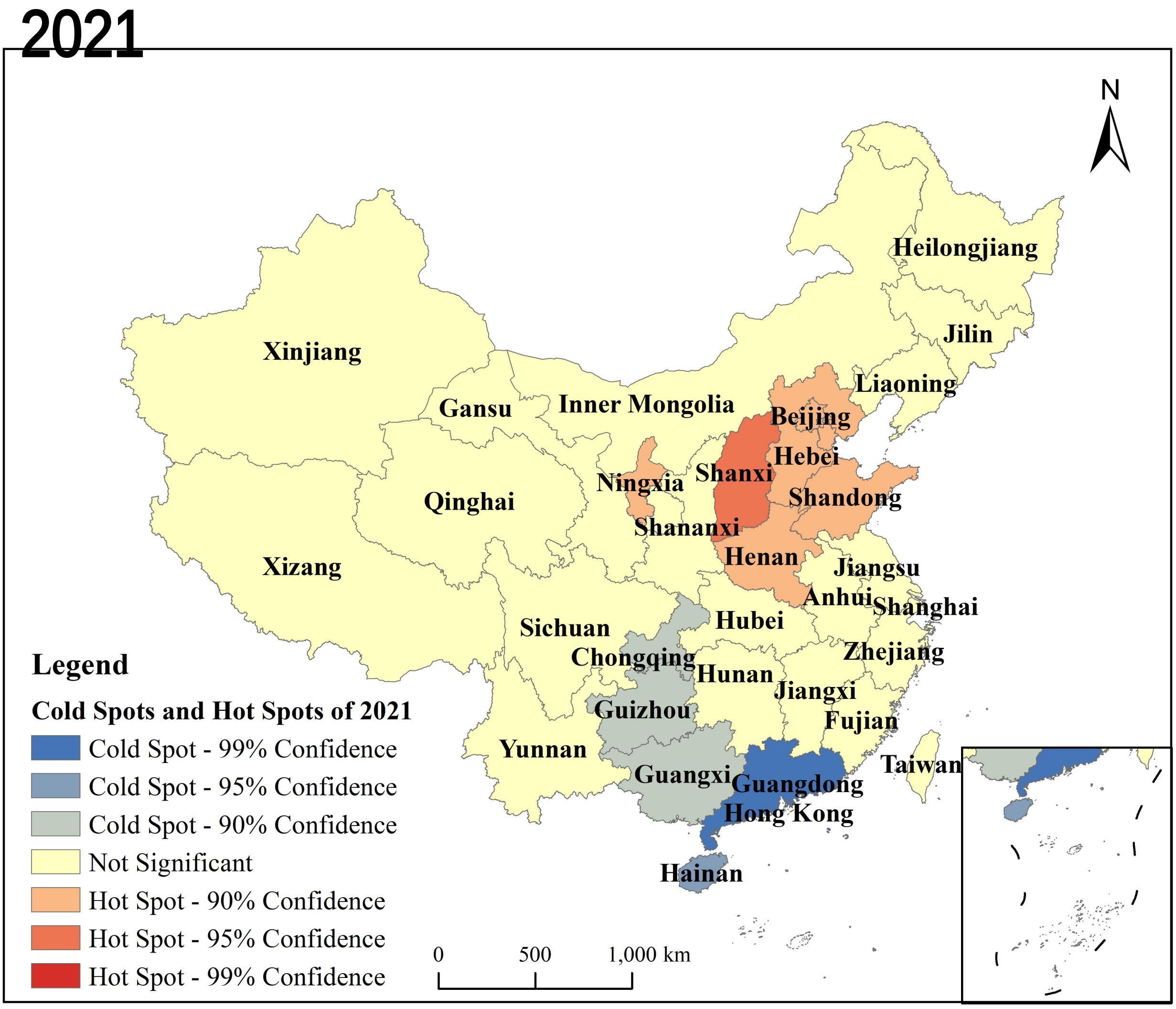

Supplementary Figure S10. Annual hotspot-coldspot map of traditional Chinese medicine (TCM) service capacity across 31 provincial-level regions in China in 2021. Local spatial clustering was identified using the Getis-Ord Gi* statistic under the economic-geographic weight matrix. Red shades indicate hot spots (high-value clusters), blue shades indicate cold spots (low-value clusters), and darker colors represent higher levels of statistical significance.

2022

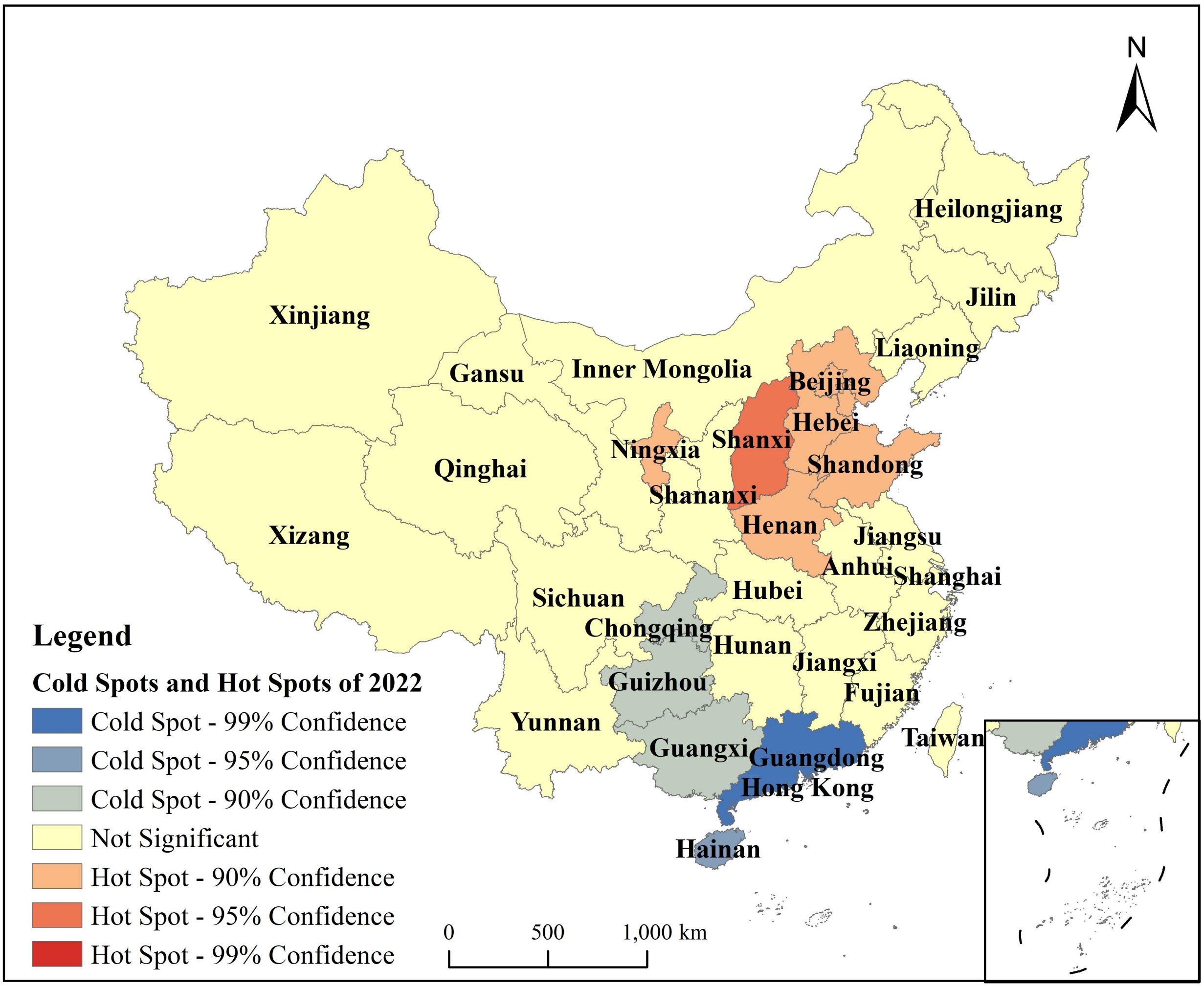

Supplementary Figure S11. Annual hotspot-coldspot map of traditional Chinese medicine (TCM) service capacity across 31 provincial-level regions in China in 2022. Local spatial clustering was identified using the Getis-Ord Gi* statistic under the economic-geographic weight matrix. Red shades indicate hot spots (high-value clusters), blue shades indicate cold spots (low-value clusters), and darker colors represent higher levels of statistical significance.

2023

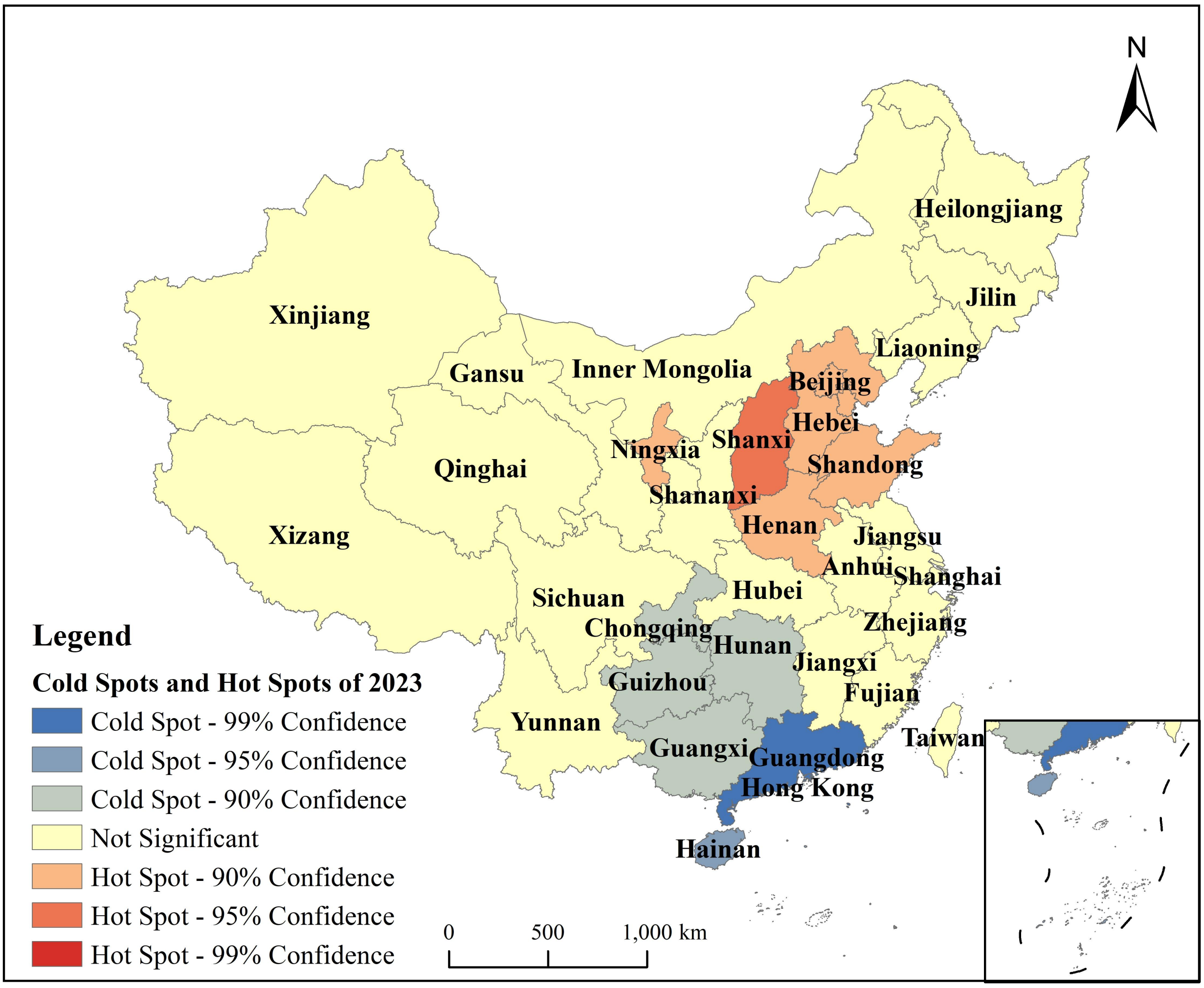

Supplementary Figure S12. Annual hotspot-coldspot map of traditional Chinese medicine (TCM) service capacity across 31 provincial-level regions in China in 2023. Local spatial clustering was identified using the Getis-Ord Gi* statistic under the economic-geographic weight matrix. Red shades indicate hot spots (high-value clusters), blue shades indicate cold spots (low-value clusters), and darker colors represent higher levels of statistical significance.
